# Molecular action of larvicidal flavonoids on ecdysteroidogenic glutathione *S*-transferase Noppera-bo in *Aedes aegypti*

**DOI:** 10.1186/s12915-022-01233-2

**Published:** 2022-02-17

**Authors:** Kazue Inaba, Kana Ebihara, Miki Senda, Ryunosuke Yoshino, Chisako Sakuma, Kotaro Koiwai, Daisuke Takaya, Chiduru Watanabe, Akira Watanabe, Yusuke Kawashima, Kaori Fukuzawa, Riyo Imamura, Hirotatsu Kojima, Takayoshi Okabe, Nozomi Uemura, Shinji Kasai, Hirotaka Kanuka, Takashi Nishimura, Kodai Watanabe, Hideshi Inoue, Yuuta Fujikawa, Teruki Honma, Takatsugu Hirokawa, Toshiya Senda, Ryusuke Niwa

**Affiliations:** 1 Graduate School of Life and Environmental Sciences, University of Tsukuba, 1-1-1 Tennodai, Tsukuba, Ibaraki 305-8572 Japan; 2 Structural Biology Research Center, Photon Factory, Institute of Materials Structure Science, High Energy Accelerator Research Organization (KEK), 1-1 Oho, Tsukuba, Ibaraki 305-0801 Japan; 3 Degree Programs in Life and Earth Sciences, Graduate School of Science and Technology, University of Tsukuba, 1-1-1 Tennodai, Tsukuba, Ibaraki 305-8572 Japan; 4 Transborder Medical Research Center, University of Tsukuba, 1-1-1 Tennodai, Tsukuba, Ibaraki 305-8575 Japan; 5 Division of Biomedical Science, Faculty of Medicine, University of Tsukuba, 1-1-1 Tennodai, Tsukuba, Ibaraki 305-8575 Japan; 6 Department of Tropical Medicine, Center for Medical Entomology, The Jikei University School of Medicine, 3-25-8 Nishishimbashi, Minato-ku, Tokyo, 105-8461 Japan; 7 Center for Biosystems Dynamics Research, RIKEN, 1-7-22 Suehirocho, Tsurumi-ku, Yokohama, 230-0045 Japan; 8 School of Pharmacy and Pharmaceutical Sciences, Hoshi University, 2-4-41 Ebara, Shinagawa-ku, Tokyo, 142-8501 Japan; 9 Drug Discovery Initiative, The University of Tokyo, 7-3-1 Hongo, Bunkyo-ku, Tokyo, 113-0033 Japan; 10 Department of Medical Entomology, National Institute of Infectious Diseases, 1-23-1 Toyama, Shinjuku-ku, Tokyo, 162-8640 Japan; 11 Institute for Molecular and Cellular Regulation, Gunma University, 3-39-15 Showa-machi, Maebashi, 371-8512 Japan; 12 School of Life Sciences, Tokyo University of Pharmacy and Life Sciences, 1432-1 Horinouchi, Hachioji, Tokyo, 192-0392 Japan; 13 Cellular and Molecular Biotechnology Research Institute, National Institute of Advanced Industrial Science and Technology, 2-4-7 Aomi, Koto-ku, Tokyo, 135-0064 Japan; 14 School of High Energy Accelerator Science, SOKENDAI University, 1-1 Oho, Tsukuba, Ibaraki 305-0801 Japan; 15 Faculty of Pure and Applied Sciences, University of Tsukuba, 1-1-1 Tennodai, Ibaraki, 305-8571 Japan; 16 Life Science Center for Survival Dynamics, Tsukuba Advanced Research Alliance (TARA), University of Tsukuba, 1-1-1 Tennodai, Tsukuba, Ibaraki 305-8577 Japan

**Keywords:** *Aedes aegypti*, Ecdysone, Ecdysteroid, Flavonoid, Glutathione *S*-transferase, Insect growth regulator, Insecticide, Mosquito, Noppera-bo

## Abstract

**Background:**

Mosquito control is a crucial global issue for protecting the human community from mosquito-borne diseases. There is an urgent need for the development of selective and safe reagents for mosquito control. Flavonoids, a group of chemical substances with variable phenolic structures, such as daidzein, have been suggested as potential mosquito larvicides with less risk to the environment. However, the mode of mosquito larvicidal action of flavonoids has not been elucidated.

**Results:**

Here, we report that several flavonoids, including daidzein, inhibit the activity of glutathione *S*-transferase Noppera-bo (Nobo), an enzyme used for the biosynthesis of the insect steroid hormone ecdysone, in the yellow fever mosquito *Aedes aegypti*. The crystal structure of the Nobo protein of *Ae. aegypti* (AeNobo) complexed with the flavonoids and its molecular dynamics simulation revealed that Glu113 forms a hydrogen bond with the flavonoid inhibitors. Consistent with this observation, substitution of Glu113 with Ala drastically reduced the inhibitory activity of the flavonoids against AeNobo. Among the identified flavonoid-type inhibitors, desmethylglycitein (4′,6,7-trihydroxyisoflavone) exhibited the highest inhibitory activity in vitro. Moreover, the inhibitory activities of the flavonoids correlated with the larvicidal activity, as desmethylglycitein suppressed *Ae. aegypti* larval development more efficiently than daidzein.

**Conclusion:**

Our study demonstrates the mode of action of flavonoids on the *Ae. aegypti* Nobo protein at the atomic, enzymatic, and organismal levels.

**Supplementary Information:**

The online version contains supplementary material available at 10.1186/s12915-022-01233-2.

## Background

Mosquitos act as vectors of many infectious diseases caused by a huge number of pathogens and parasites, epitomized by the spread of malaria [1]. Despite decades of intensive research, the effective and sustainable management of mosquito vector populations remains a difficult challenge [2, 3]. Among vector mosquito species, the mosquitos of the genus *Aedes*, which includes the yellow fever mosquito *Ae. aegypti*, are competent vectors of several human infectious viruses, such as the dengue virus, yellow fever virus, and Zika virus. As the *Aedes* mosquitos are widely distributed, they are recognized as an important factor in the global burden of infectious diseases.

Many insecticides have been developed and applied for the control of *Aedes* vectors [2, 4]. However, emergence of resistance in wild *Ae. aegypti* populations has reduced the efficiency of insecticides [4–8]. For example, although pyrethroids and organophosphates are the most widely used and effective insecticides against *Ae. aegypti*, resistance to these insecticides has been reported [4]. In the case of pyrethroid resistance, mutations in a voltage-gated sodium channel gene have been shown to induce a type of resistance known as the *knockdown resistance*, which has been globally observed in the *Ae. aegypti* populations [4]. Therefore, a new insecticide, whose chemical structure and target differ from those of the currently used insecticides, is highly desirable.

Flavonoids, which are secondary metabolites from plants and other microorganisms that can affect many aspects of insect development and physiology [9], can exert larvicidal activity against *Ae. aegypti* [10]. For example, flavonoid extracts or purified flavonoids from several plants exhibit larvicidal activity against *Ae. aegypti* and other vector mosquitos [11–16]. Culture broths of a species of the actinomycete *Streptomyces* show toxicity to *Ae. aegypti* larvae, and this was revealed to be due to several flavonoids, including genistein and daidzein [17]. It is generally expected that flavonoids are relatively safe, showing less risk to the environment with minimal impacts on animal and human health, and are thought to be beneficial to human health [18–20]. Therefore, flavonoids could be preferable lead compounds for developing an environment-friendly insecticide to control *Ae. aegypti* [21, 22]*.* However, the underlying mechanism of action of these flavonoids at the molecular, cellular, and organismal levels remains largely unknown. Although some flavonoids are known to inhibit acetylcholine esterase activity, no correlation is found between larvicidal activity and acetylcholine esterase inhibition [14]. It is important to understand the modes of action of the flavonoids on larvicidal activity against *Ae. aegypti* for the development of safe and biorational flavonoidal insecticides for future resistance management.

Here, we report that some flavonoids act as the inhibitors of the *Ae. aegypti* Noppera-bo (AeNobo) protein, which belongs to the glutathione *S*-transferase (GST) epsilon subfamily. Nobo plays a specialized role in the biosynthesis of the principal insect steroid hormones, ecdysteroids [23–25]. Similar to other ecdysteroidogenic enzymes [26], genetic studies have demonstrated that Nobo is required for molting and metamorphosis (i.e., ecdysteroid-dependent developmental processes) in the fruit fly *Drosophila melanogaster* and the silkworm *Bombyx mori* [23–25]. As ecdysteroids are particularly important for the life cycle of insects, it is expected that a chemical inhibitor of ecdysteroidogenic enzymes, including Nobo, would be an insect growth regulator (IGR) that impacts insect development without affecting organisms other than insects [27, 28]. Our group has developed a high-throughput screening system to identify chemical compounds that inhibit in vitro enzymatic activity of recombinant purified Nobo proteins [29]. Using this system, we previously isolated multiple inhibitors of *D. melanogaster* Nobo (DmNobo) and succeeded in an integrated structure biological analysis to partly reveal the mode of action of these inhibitors, including the vertebrate female sex hormone 17β-estradiol [30, 31]. In this study, we expanded our strategy to the AeNobo recombinant protein to identify potential AeNobo inhibitors and demonstrate their mode of action.

## Results

### Identification of several flavonoids as AeNobo inhibitors

According to a Basic Local Alignment Search Tool database search, an *Ae. aegypti* gene closest to the *D. melanogaster nobo* gene is *LOC5569853*, annotated by the *Ae. aegypti* genome database (Locus tag: AaeL_AAEL007955). The GenBank data Accession number XM_001658698.3 predicts that *LOC5569853* encodes a protein having 271 amino acid (aa) residues, while another GenBank database EAT40301.1 predicts that *LOC5569853* encodes a 220-aa protein. We realized that the 271-aa protein has a long extension at the N-terminus as compared to the 220-aa protein and DmNobo (Additional file 1: Figure S1*A*), and the 220-aa protein (instead of the 271-aa protein) is substantially similar to DmNobo in terms of aa length. Subsequently, we examined whether a short *LOC5569853* cDNA encoding the 220-aa protein can compensate for *DmNobo* loss-of-function mutation during development. The forced expression of *DmNobo* driven by *phantom-GAL4* fly strain rescues developmental lethality of *DmNobo* knock-out homozygous mutant animals [23]. We found that *phantom-GAL4*-driven expression of the short cDNA allowed *DmNobo* knock-out homozygous mutant animals to complete their development and grow to the adult stage (Additional file 2: Table S1). This result confirmed that *LOC5569853* is an *Ae. aegypti* ortholog of *nobo*, and the 220-aa protein is functionally equivalent to DmNobo. Hereafter, we designate *LOC5569853* as *AeNobo.*

Next, we examined whether AeNobo enzymatic activity was inhibited by 17β-estradiol, which we previously identified as a DmNobo inhibitor (Additional file 1: Figure S2*A*). We prepared a purified AeNobo recombinant protein (220-aa length) using an *Escherichia coli* protein expression system. The GST enzymatic activity was examined using the fluorogenic artificial substrate 3,4-dinitrobenzamidedichlorofluorescein (3,4-DNADCF) [29]. In this assay system, GSTs catalyze GSH conjugation to the weakly fluorescent molecule, 3,4-DNADCF, giving rise to a highly fluorescent product, 4-GS-3-NADCF. Using this system, we found that the specific enzymatic activity of AeNobo to conjugate glutathione (GSH) and 3,4-DNADCF was 2.04 ± 0.04 μmol·min^−1^·mg^−1^ in vitro, suggesting that AeNobo exhibits GST enzymatic activity. However, we discovered that the concentration of 50% inhibition (IC_50_) of 17β-estradiol against AeNobo was 21.3 μM, which was approximately 10-fold higher than that against DmNobo (1.2–2.3 μM) (Additional file 1: Figure S2*B*) [29, 30]. These data motivated us to identify other chemical compounds that inhibit AeNobo enzymatic activity, having IC_50_ values equivalent to or lower than that of 17β-estradiol against DmNobo.

**Fig. 1:**
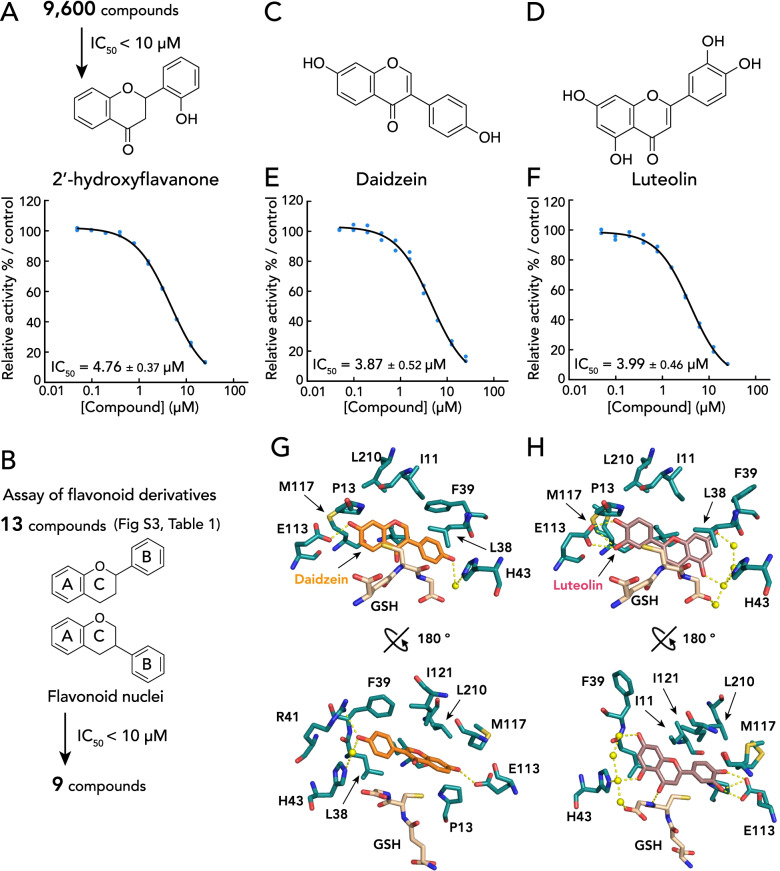
Identification and characterization of daidzein and luteolin as flavonoids that inhibit the AeNobo enzymatic activity and interact with H-sites of AeNobo. **A** Schematic of the library screen to identify chemical compounds that inhibit AeNobo in vitro with IC_50_ values of less than 10 μM. One of the identified compounds was 2′-hydroxyflavanone. **B** Schematic of a screen to identify flavonoid compounds that inhibit AeNobo in vitro with IC_50_ values of less than 10 μM. The IC_50_ values of the nine tested compounds, including daidzein and luteolin, were less than 10 μM. **C**, **D** Chemical structures of daidzein (**C**) and luteolin (**D**). **E**, **F** Inhibition of the GSH conjugation activities of AeNobo with an artificial fluorescent substrate, 3,4-DNADCF, in the presence of daidzein (**E**) and luteolin (**F**). Each relative activity is defined as the ratio of activity compared between the respective proteins without the flavonoids. All the data points in duplicate assays are indicated. **G**, **H** Amino acid residues interacting with daidzein (**G**) and luteolin (**H**). Carbon atoms of daidzein and luteolin are colored orange and light violet, respectively. Oxygen, nitrogen, and sulfur atoms are colored red, blue, and yellow, respectively. A water molecule interacting with each ligand is represented with a yellow sphere. Amino acid residues located within a 4.0-Å radius of the nearest atom of the flavonoids are shown. Additionally, amino acid residues that form hydrogen bonds within a 3.3-Å radius of the nearest atom of the flavonoids are also shown. Hydrogen bonds are illustrated by dashed yellow lines. The two views are related by a 180° rotation around the bold black line axis

To identify AeNobo inhibitors, we performed high-throughput screening for the inhibitors of GSH conjugation activity of AeNobo using 3,4-DNADCF. Among 9600 chemical compounds obtained from the Drug Discovery Initiative of the University of Tokyo [29], we identified 2′-hydroxyflavanone with an IC_50_ value of 4.76 μM (Fig. 1A). Based on this result, we focused on flavonoids because flavonoids are well known to affect several aspects of the insect life cycle [9]. To further determine the kind of flavonoid compounds that inhibit AeNobo enzymatic activity, we examined 13 flavonoids excluding 2′-hydroxyflavanone, which we easily obtained at a relatively lower price in Japan, for the in vitro enzymatic activity assay. The 13 selected flavonoids included flavanones, flavone, isoflavones, flavanols, isoflavan, and anthocyanidins (Table 1, Additional file 1: Figure S3). We found that the IC_50_ values of 9 compounds, including luteolin, biochanin A, daidzein, fisetin, kaempferol, myricetin, quercetin, cyanidin chloride, and petunidin were lower than 10 μM (Fig. 1B, Table 1). These results suggest that some, but not all, flavonoid chemicals can be classified as AeNobo inhibitors.

**Table 1:** Inhibitory activity of 2′-hydroxyflavanone and other 13 flavonoids against AeNobo. Fifteen flavonoids, including the subclasses of flavonone, flavone, isoflavone, flavonol, isoflavan, and anthocyanidin, are illustrated in Additional file 1: Figure S3. “No inhibition” means that the IC_50_ value of a compound is greater than 25 μM. Among the examined chemicals, biochanin A is the only estrogenic chemical that inhibits the in vitro enzymatic activity of AeNobo. s.d. standard deviation, - not determined

Compound	Subclass	IC_50_ to AeNobo-WT (μM) (mean ± s.d.)	IC_50_ to AeNobo-E113A (μM) (mean ± s.d.)
2′-Hydroxyflavanone	Flavanone	4.76 ± 0.37	No inhibition
Naringenin	Flavanone	No inhibition	-
Luteolin	Flavone	3.99 ± 0.46	35.8 ± 5.73
Biochanin A	Isoflavone	1.84 ± 0.06	No inhibition
Daidzein	Isoflavone	3.87 ± 0.52	No inhibition
(+) Catechin hydrate	Flavonol	No inhibition	-
Fisetin	Flavonol	1.69 ± 0.04	No inhibition
Kaempferol	Flavonol	4.91 ± 1.69	No inhibition
Myricetin	Flavonol	2.91 ± 0.27	No inhibition
Quercetin	Flavonol	0.963 ± 0.094	No inhibition
Tamarixetin	Flavonol	No inhibition	-
*S*-equol	Isoflavan	No inhibition	-
Cyanidin chloride	Anthocyanidin	2.15 ± 0.24	No inhibition
Petunidin	Anthocyanidin	14.9 ± 2.01	No inhibition

### Non-flavonoidal estrogenic compounds do not inhibit AeNobo

Many flavonoids, including daidzein and kaempferol [32], are known to act as agonists of the estrogen receptor [33–35]. Therefore, we wondered whether non-flavonoidal estrogenic compounds can also inhibit AeNobo enzymatic activity in vitro. Consequently, we examined 18 non-flavonoidal estrogenic compounds, including well-known environmental disruptors such as bisphenol A and diethylstilbestrol, for the in vitro enzymatic assay using 3,4-DNADCF. Our results revealed that all the non-flavonoidal estrogenic compounds examined in this study failed to inhibit AeNobo in vitro (IC_50_ > 25 μM) (Additional file 2: Table S2), suggesting that estrogenic activity is not a prerequisite for a compound to be classified as an AeNobo inhibitor.

### Binding mode of flavonoids to AeNobo

To reveal the molecular mechanism through which these flavonoids inhibit AeNobo enzymatic activity, we conducted an X-ray crystallographic analysis of AeNobo. Gel filtration chromatography revealed that AeNobo forms a homodimer in solutions (Additional file 1: Figure S4*A*), suggesting that its dimeric structure is a biological unit similar to DmNobo and other canonical GSTs [30, 31, 36]. Subsequently, we determined the crystal structure of the AeNobo protein in the presence of GSH at 1.51 Å resolution by the molecular replacement method using the structure of DmNobo (PDB ID: 6KEM) [37] as an initial model (Additional file 1: Figure S4*B*). We found that the asymmetric unit of AeNobo is composed of four chains: A, B, C, and D. Each chain forms a biological dimer with a symmetry-related subunit by a crystallographic twofold axis (Additional file 1: Figure S4*C* and S4*D*). AeNobo adopts a canonical GST fold (Additional file 1: Figure S4*D*), which has a well-conserved GSH-binding site (G-site) and a hydrophobic substrate-binding pocket (H-site) adjacent to the G-site (Additional file 1: Figure S5).

Next, crystal structures of AeNobo-GSH complexed with daidzein (AeNobo-GSH-daidzein) and luteolin (AeNobo-GSH-luteolin) were determined at 1.95 Å and 1.50 Å resolution, respectively (Additional file 2: Table S3). Daidzein and luteolin inhibited AeNobo enzymatic activity with an IC_50_ value of 3.87 μM and 3.99 μM, respectively (Fig. 1C, D); these compounds are known to exhibit larvicidal activity toward *Ae. aegypti* [17, 38]. The subunit structures of AeNobo complex with inhibitors were essentially the same as those of the substrate-free form (Additional file 2: Table S4). Electron densities of the inhibitors were observed in all four chains (Additional file 1: Figure S5). Interactions between AeNobo and GSH in the G-site were essentially the same in the presence or absence of daidzein (Additional file 1: Figure S6*A*) or luteolin (Additional file 1: Figure S6*B*), suggesting that these flavonoids do not interfere with the interaction between AeNobo and GSH.

Although daidzein and luteolin both bind to the AeNobo H-site, the binding orientations of daidzein and luteolin in the H-site are opposite to each other. The A-ring of daidzein is nested deep inside the H-site, but the A-ring of luteolin is located at the entrance of the H-site (Fig. 1E, F). Nevertheless, AeNobo uses the same aa residues to interact with daidzein and luteolin directly or indirectly via water molecules. For example, these inhibitors interact with Leu-38, His-43, and Glu-113. Additionally, they exhibit hydrophobic interactions with Ile-11, Pro-13, Leu-38, Met-117, Ile-121, and Leu-210 (Fig. 1E, F).

### The interaction between Glu-113 of AeNobo and flavonoid inhibitors is essential for inhibition

**Fig. 2:**
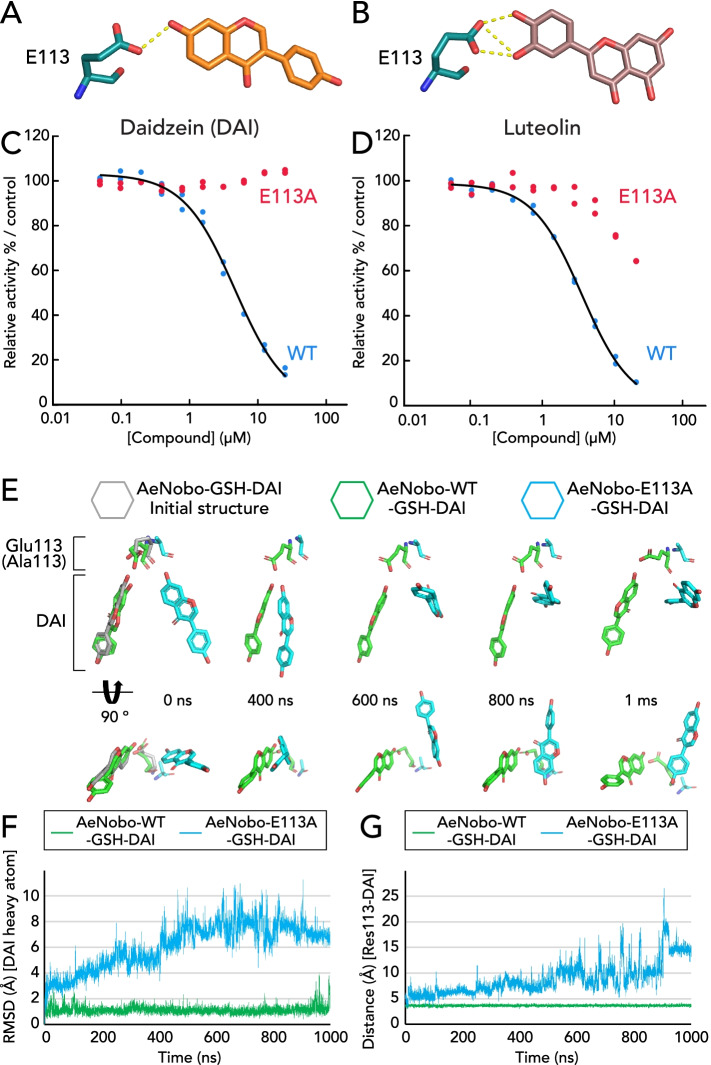
Glu-113 is essential for the binding of AeNobo to daidzein and luteolin. **A**, **B** The hydrogen bonds between Glu-113 and the hydroxyl residues of C7 of the A-ring of daidzein (DAI; **A**) and of C3′ and C4′ of the B-ring of luteolin (**B**) are highlighted from Fig. 1**G** and **H**, respectively. Carbon atoms of daidzein and luteolin are colored orange and light violet, respectively. Oxygen, nitrogen, and sulfur atoms are colored red, blue, and yellow, respectively. **C**, **D** Inhibition of the GSH conjugation activities of wild-type AeNobo (WT, blue dots and black solid curves) and the mutated AeNobo substituting Glu-113 with Ala (E113A, red dots) using 3,4-DNADCF in the presence of DAI (**C**) and luteolin (**D**). Each relative activity is defined as the ratio of activity compared between the respective proteins without the flavonoids. All the data points in duplicate assays are indicated. **E**, **F** In silico evaluation of the contribution of Glu-113 to the interaction between AeNobo and DAI. MD simulations of the AeNobo-WT or AeNobo-E113A complex with GSH and DAI in a SPC-water model were conducted at 300 K for 1000 ns. These simulations were performed in triplicate. **E** MD models at several representative time points of AeNobo-WT and AeNobo-E113A with DAI. The lower models are rotated 90° from the upper models. **F** RMSD of DAI heavy atoms in the MD simulations. Green: WT; blue: E113A mutation model. **G** Distance between the carboxylate C atom of Glu-113 of AeNobo-WT or Cβ of Ala-113 of AeNobo-E113A and the O7 atom of DAI at each frame

Among the aa residues that interact with daidzein and luteolin, we focused on Glu-113. The Oε of Glu-113 forms a hydrogen bond with the hydroxyl group at C7 of daidzein and two hydroxyl groups at C3′ and C4′ of luteolin (Figs. 1G, H, and 2A, B). The hydrogen bonds were observed in all 4 chains (Additional file 1: Figure S7*A*, *B*). Glu-113 of AeNobo corresponds to Asp-113 of DmNobo [30], as supported by the superposition of the two structures (Additional file 1: Figure S1*B*). The interaction between Asp-113 of DmNobo and a hydroxyl group of 17β-estradiol is essential for the inhibition, as 17β-estradiol does not inhibit the enzymatic activity of the point mutated DmNobo protein in which Asp-113 is substituted with Ala [30]. As Glu has a carboxyl group in the side chain similar to Asp, we speculated that Glu-113 of AeNobo also has a significant impact on the inhibitory activities of daidzein and luteolin.

The importance of the Glu-113–flavonoid hydrogen bond for flavonoid binding was biochemically examined with a recombinant mutated AeNobo protein carrying an E113A aa substitution (AeNobo-E113A). While AeNobo-E113A retains GST activity with specific enzymatic activity for the conjugation of GSH and 3,4-DNADCF of 1.41 ± 0.08 μmol·min^−1^·mg^−1^ in vitro, the enzymatic activity of AeNobo-E113A was not inhibited by any flavonoid, including daidzein and luteolin at a concentration of 25 μM (Table 1 and Fig. 2C, D). Moreover, molecular dynamics (MD) simulations showed that daidzein easily dissociated from the AeNobo-E113A with an average root mean square deviation (RMSD) of 6.13 Å, while daidzein remained stable in the H-site of wild-type AeNobo (AeNobo-WT) with an average RMSD of 1.13 Å (Fig. 2E, F, G, Additional file 1: Figure S8, Additional file 3: Movie 1, Additional file 4: Movie 2). These data suggest that the hydrogen bonding between Glu-113 and daidzein is essential for the inhibitory activity of the flavonoids against AeNobo.

### The hydroxyl groups of flavonoids that form the hydrogen bond with AeNobo are essential for inhibiting AeNobo

**Fig. 3:**
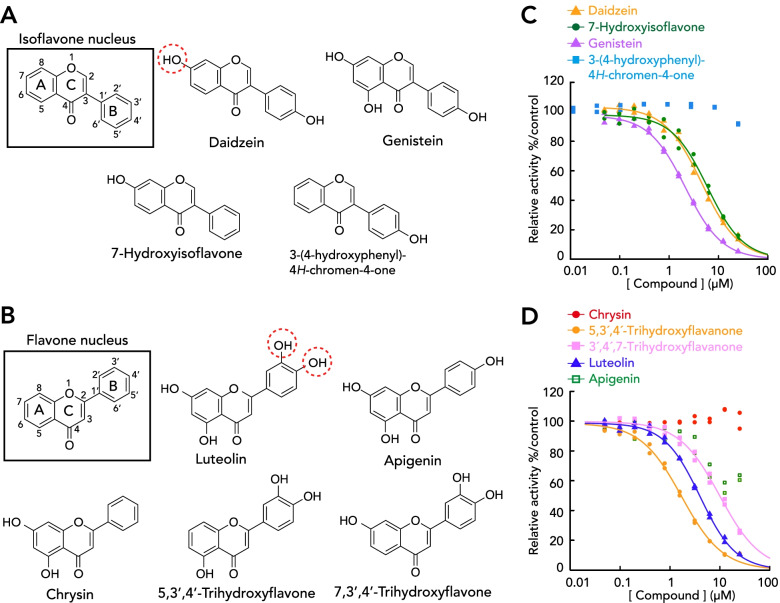
Structure-activity relationship of flavonoid derivatives exhibiting inhibitory activities against AeNobo. **A**, **B** Chemical structures of daidzein derivatives (**A**) and luteolin derivatives (**B**), which possess isoflavone and flavone nuclei, respectively. Red dashed circles indicate the hydroxyl residues that form hydrogen bonds with Glu-113 of AeNobo. **C**, **D** Inhibition of the GSH conjugation activities of AeNobo using 3,4-DNADCF in the presence of daidzein derivatives (**C**) and of luteolin derivatives (**D**). Each relative activity is defined as the ratio of activity compared between the respective proteins without the flavonoids. All the data points in duplicate assays are indicated

To further evaluate the significance of the hydrogen bond between Glu-113 of AeNobo and the flavonoids, we took another approach to utilize several chemical derivatives of daidzein and luteolin. Each chemical derivative lacked one or more hydroxyl group(s) on its flavonoidal carbon structure as compared to daidzein and luteolin (Fig. 3A, B).

We found that 3-(4-hydroxyphenyl)-4*H*-chromen-4-one, which lacks a hydroxyl group at C7 compared to daidzein (Fig. 3A, Table 2), did not inhibit the enzymatic activity of AeNobo (Fig. 3C, Table 2). In contrast, 7-hydroxyisoflavone, which lacks a hydroxyl group at C4′ compared to daidzein (Fig. 3A, Table 2), retained inhibitory activity against AeNobo (Fig. 3C, Table 2). These data suggest that the hydroxyl group at C7 of daidzein is critical for the inhibition.

**Table 2:** Inhibitory activity of daidzein derivatives against AeNobo. Daidzein derivatives used in this study are illustrated in Fig. 3A. Besides IC_50_ values, this table shows the presence of the hydroxyl residues (-OH) in the carbon positions of isoflavone nuclei. For example, luteolin possesses the hydroxyl residues at C3′, C4′, C5, and C7 position of the isoflavone nuclei. “No inhibition” means that an IC_50_ value of a compound is larger than 25 μM. s.d. standard deviation

Compound	C4′	C5	C6	C7	IC_50_ (μM) (mean ± s.d.)
Daidzein	-OH			-OH	3.87 ± 0.52
Genistein	-OH	-OH		-OH	1.86 ± 0.41
7-Hydroxyisoflavone				-OH	5.61 ± 0.30
3-(4-Hydroxyphenyl)-4*H*-chromen-4-one	-OH				No inhibition
Desmethylglycitein	-OH		-OH	-OH	0.293 ± 0.012

In the case of luteolin derivatives, apigenin, which lacks a hydroxyl group at C3′ (Fig. 3B, Additional file 2: Table S5), exhibited a substantial decrease in its inhibitory activity having an IC_50_ value >25 μM (Fig. 3D, Additional file 2: Table S5). Moreover, chrysin, which lacks two hydroxyl groups at C3′ and C4′ (Fig. 3B, Additional file 2: Table S5), did not exhibit any inhibitory activity (Fig. 3D, Additional file 2: Table S5). In contrast, 7,3′4′-trihydroxyflavone and 5,3′4′-trihydroxyflavone, both of which are luteolin derivatives but lack hydroxyl groups at C5 and C7, respectively (Fig. 3B, Additional file 2: Table S5), retained inhibitory activity against AeNobo to a level comparable to that of luteolin (Fig. 3D, Additional file 2: Table S5). These data suggest that the hydroxyl groups at C3′ and C4′ of luteolin are critical for the inhibition. Furthermore, the hydroxyl group at C7 of daidzein and the two hydroxyl groups of C3′ and C4′ of luteolin form hydrogen bonds with Glu-113 of AeNobo. Therefore, these results also support our hypothesis that the interaction between Glu-113 and the flavonoids is crucial for the inhibition.

### Desmethylglycitein is an efficient flavonoidal inhibitor of AeNobo

**Fig. 4:**
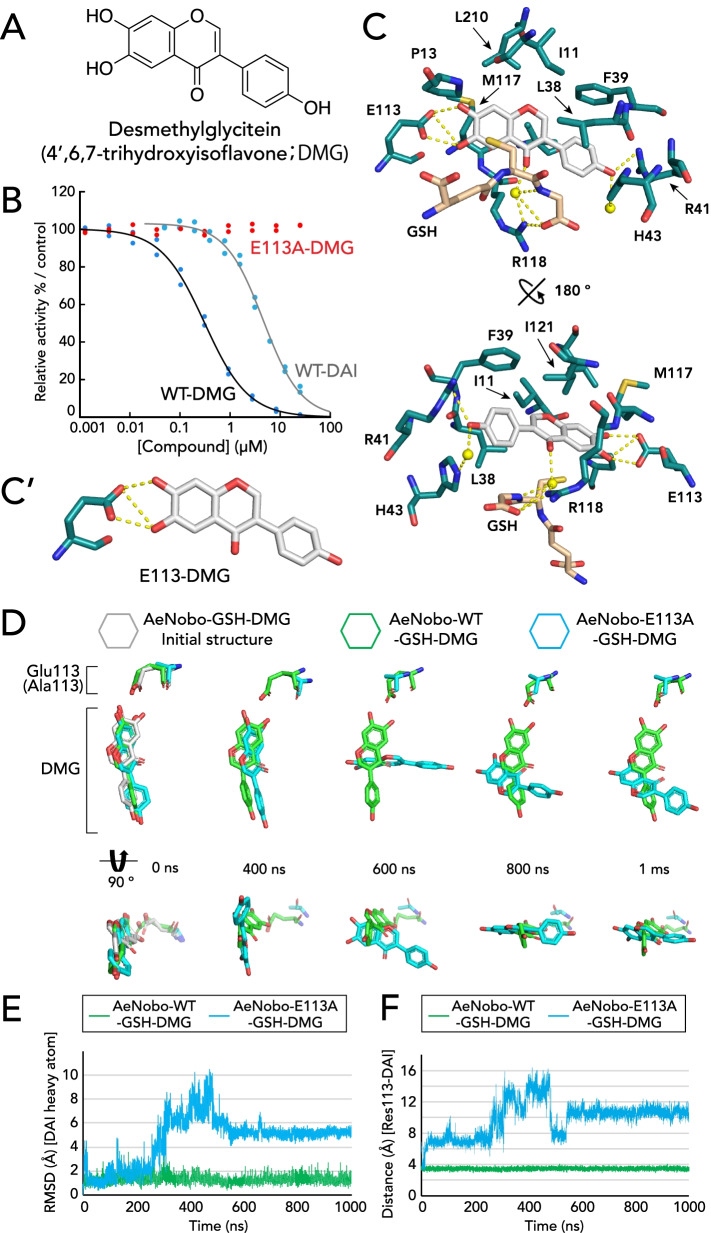
Desmethylglycitein (DMG) inhibits AeNobo. **A** Chemical structures of DMG, also known as 4′,6,7-trihydroxyisoflavone. **B** Inhibition of the GSH conjugation activities of wild-type AeNobo (WT, blue dots and black solid curves) and the mutated AeNobo substituting Glu-113 with Ala (E113A, red dots) using 3,4-DNADCF in the presence of DMG. Each relative activity is defined as the ratio of activity compared between the respective proteins without DMG. All of the data points in duplicate assays are indicated. **C** Amino acid residues interacting with DMG. Carbon atoms of DMG are colored gray. Oxygen, nitrogen, and sulfur atoms are colored red, blue, and yellow, respectively. A water molecule interacting with each ligand is represented with a yellow sphere. Amino acid residues located within a 4.0-Å radius of the nearest atom of the flavonoids are shown. Additionally, amino acid residues that form hydrogen bonds within a 3.3-Å radius of the nearest atom of the flavonoids are also shown. Hydrogen bonds are illustrated by dashed yellow lines. The two views are related by a 180° rotation around the bold black line axis. Note that the hydrogen bond interaction between the hydroxyl residue of the B-ring and Arg-41 in chain D is indicated in this figure, while the direct interaction between DMG and Arg-41 is not observed in chains A, B, or C. **C′** The hydrogen bonds between Glu-113 and the hydroxyl residues of C6 and C7 of the A-ring of DMG are highlighted. **D**, **E** In silico evaluation of the contribution of Glu-113 to the interaction between AeNobo and DMG. MD simulations of the AeNobo-WT or AeNobo-E113A complex with GSH and DMG in a SPC-water model were conducted at 300 K for 1000 ns. These simulations were performed in triplicate. **D** MD models at several representative time points of AeNobo-WT and AeNobo-E113A with DAI. The lower models are rotated 90° from the upper models. **E** RMSD of DMG heavy atoms in the MD simulations. Green: WT; blue: E113A mutation model. **F** Distance between the carboxylate’s C atom of Glu-113 of AeNobo-WT or Cβ of Ala-113 of AeNobo-E113A and the O6 or O7 atom of DMG at each frame. The nearest distances between O6 and O7 atoms are represented in this graph

As described above, our crystal structure analysis on luteolin revealed that Glu-113 interacts with two hydroxyl groups. In contrast, daidzein possesses one hydroxyl group that forms a hydrogen bond with Glu-113. Therefore, we hypothesized that daidzein derivatives that possess an additional hydroxyl group on the A-ring show stronger interactions with Glu-113 and thus exhibit more efficient inhibitory activities against AeNobo than daidzein. To test this hypothesis, we utilized genistein (Fig. 3A) and desmetylglycitein (DMG) (Fig. 4A), which have an additional hydroxyl group at C5 and C6, respectively, compared to daidzein. We found that genistein exhibited inhibitory activity against AeNobo with an IC_50_ value of 1.86 μM (Fig. 3C and Table 2), which is not very different from that of daidzein (Table 2). In contrast, DMG displayed the highest inhibitory activity against AeNobo among the tested flavonoids; the IC_50_ value of DMG was 0.287 μM, the lowest among all flavonoid inhibitors that were examined in this study (Fig. 4B and Table 2).

Next, we determined the crystal structure of AeNobo-WT complexed with GSH and DMG (AeNobo-GSH-DMG). We found that DMG interacts with the H-site of AeNobo (Fig. 4C) in a manner very similar to daidzein (Fig. 1G, H, Additional file 1: Figure S6*A*), except that Arg-118 indirectly interacts with the ketone group of the C-ring of DMG via a water molecule (Fig. 4C). Furthermore, as expected, DMG has two hydroxyl groups at C6 and C7 of A-ring, both of which form hydrogen bonds with Oε of Glu-113 of AeNobo (Fig. 4C, C′). The hydrogen bonds were observed in all 4 chains (Additional file 1: Figure S7*C*). The hydrogen bond between Glu-113 and DMG is essential to its inhibitory activity; the enzymatic activity of AeNobo-E113A was not inhibited by DMG even at a concentration of 25 μM (Fig. 4B, Table 2). MD simulations also demonstrated that DMG easily dissociated from the AeNobo-E113A with an average RMSD of 4.59 Å, while DMG remained stable in the H-site of AeNobo-WT with an average RMSD of 1.32 Å (Fig. 4D, E, Additional file 1: Figure S8, Additional file 5: Movie 3, Additional file 6: Movie 4). These results suggest that, similar to daidzein, the hydrogen bonding between Glu-113 and DMG is crucial for stable binding of DMG to the H-site.

As described above, the inhibitory activity of DMG is stronger than that of luteolin. However, both DMG and luteolin utilize two hydroxyl groups of the A-ring to form the hydrogen bonds with Oε of Glu-113 of AeNobo. In this situation, the stronger inhibitory activity of DMG than that of luteolin cannot be explained only by the interaction with Glu-113. To further understand the action of DMG on AeNobo, we analyzed structural differences between AeNobo-GSH-luteolin and AeNobo-GSH-DMG. We realized that the structural difference between the luteolin and DMG complexes lies in the presence/absence of a CH-π interaction with Phe-39. We observed a CH-π interaction between Phe-39 and the B-ring of DMG (Additional file 1: Figure S9*A*), but not the A-ring of luteolin (Additional file 1: Figure S9*B*). This difference arose due to the opposite orientation of DMG as compared to luteolin, as the A-ring of DMG and the B-ring of luteolin were near Glu-113 of AeNobo. As the hydroxyl residue at C7 of luteolin is close to Phe-39, the A-ring of luteolin cannot form a CH-π interaction with Phe-39. This observation raises the possibility that the two hydrogen bonds as well as the CH-π interaction contribute to the inhibitory properties of DMG. The importance of the CH-π interaction between DMG and Phe-39 was confirmed using the AeNobo-F39L variant. While the variant retained GST activity with a specific enzymatic activity of 1.85 ± 0.26 μmol·min^−1^·mg^−1^, the inhibitory activity of DMG was reduced compared to that of AeNobo-WT, showing an IC_50_ value of 1.16 μM (Additional file 1: Figure S9*C*). In contrast, the IC_50_ value of luteolin to AeNobo-F39L was 1.58 μM (Additional file 1: Figure S9*D*), which is comparable to the inhibitory activity against AeNobo-WT. These data suggest that the CH-π interaction with Phe-39 contributes to the inhibitory activity of DMG.

### Desmethylglycitein suppresses Ae. aegypti larval development

**Fig. 5:**
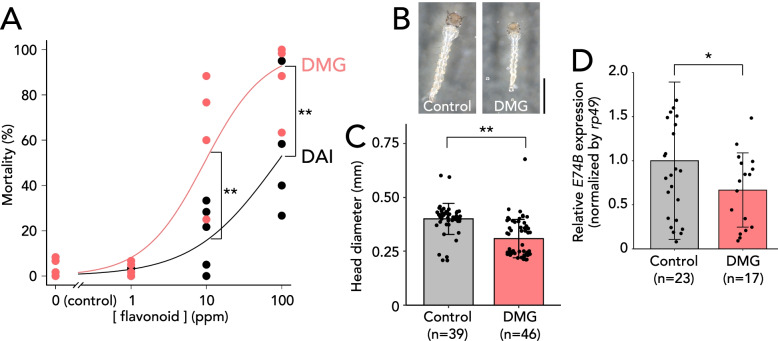
Larvicidal activity of desmethylglycitein (DMG) on *Ae. aegypti.* **A** Survival rates of the 1st instar larvae of *Ae. aegypti* 24 h after treatments of daidzein (DAI, black dots and lines) and DMG (magenta dots and lines). Control (0.1% ethanol without any flavonoids), and 1, 10, and 100 ppm of flavonoids were used. Each dot represents survival rates of twenty larvae in each independent experiment. Fitting curves were generated using log-logistic equation: Mortality = 1/(1+exp{*b*(log([flavonoid concentration])-log(LD_50_)}); *b* = −0.781911 and LD_50_ = 85.83 ppm for DAI; *b* = −1.081635 and LD_50_ = 9.39 ppm for DMG. **p < 0.01 Student’s *t*-test. **B** Representative photos of control and 2.5 ppm DMG-treated *Ae. aegypti* larvae 24 h after treatments. Scale bar, 1 mm. **C** The transverse diameter of the *Ae. aegypti* larval head was measured 24 h after adding 2.5 ppm DMG or control 0.1% DMSO. Raw data are described in Additional file 2: Table S6. ** *p* < 0.01 Student’s *t*-test. Error bars: standard deviations. **D** RT-qPCR analysis of *E74B* mRNA level under the conditions described for **C**. *E74B* mRNA levels are normalized by *rp49* mRNA levels. A mean normalized expression level of *E74B* is set as 1. *p < 0.05 Student’s *t*-test. Error bars: standard deviations

Daidzein and genistein exhibit larvicidal activity against *Ae. aegypti* [17]. As DMG is a stronger inhibitor of AeNobo than daidzein and genistein, we expected that DMG is a more efficient larvicidal reagent than daidzein and genistein. To test this, we conducted larvicidal assays using *Ae. aegypti* larvae. Three hours after hatching, we placed *Ae. aegypti* 1st instar larvae in water containing 1–100 ppm of daidzein or DMG in 0.1% EtOH and then counted the number of living larvae 24 h after the treatment. Based on our experimental conditions, 50% lethal dose (LD_50_) of DMG was observed to be approximately 9.39 ppm, while daidizen, which has been reported as a larvicide for *Ae. aegypti* [17], exhibited a LD_50_ of 85.8 ppm (Fig. 5A). These results suggest that the inhibitory activities of the flavonoids correlated with their larvicidal activity.

We further examined the effect of DMG on *Ae. aegypti* 1st instar larvae in more detail. In these experiments, we utilized 2.5 ppm DMG as this concentration did not lead to significant lethality (Fig. 5A) and therefore avoided an artifact of the lethality. First, we identified the larval instars by measuring the transverse diameter of the *Ae. aegypti* larval head 24 h after the treatment. A previous study described the head diameter of the 1st instar to be approximately 0.3 mm or less, while that of the mature 2nd instar was approximately 0.45 mm [39]. Using this indicator, we found that a head diameter of 2.5 ppm DMG-treated larvae was significantly smaller than that of the control larvae (Fig. 5B, C, Additional file 2: Table S6). More specifically, the head diameter was less than 0.3 mm in 30 of 57 DMG-treated larvae, however only four of the 50 control larvae (Additional file 2: Table S6). These results suggest that DMG treatment leads to retarded development at the 1st instar. Furthermore, in these animals, the mRNA level of *E74B*, an ecdysone-inducible gene [40], tended to be reduced in DMG-treated larvae when compared to control larvae (Fig. 5D), which was consistent with the expected effect of DMG on ecdysteroid biosynthesis.

We also conducted larvicidal assays using *D. melanogaster*. When we placed *D. melanogaster* 1st instar larvae in food containing 10 ppm DMG, pupariation rate and pupation timing were not affected when compared with a control group (Additional file 1: Figure S10). Furthermore, most larvae became adults. This result suggests that to some extent DMG exhibits a target species selectivity.

Taken together, we identified DMG as the most active flavonoidal larvicide discovered to date that suppresses *Ae. aegypti* larval development.

## Discussion

In this study, we showed that several flavonoids, including genistein, luteolin, and DMG, inhibit AeNobo enzymatic activity. Additionally, our X-ray crystallographic analysis and MD simulation revealed that Glu-113 and Phe-39 of AeNobo, particularly the former, are crucial for the interaction between AeNobo and flavonoids. Consistent with this observation, the point-mutated AeNobo proteins, such as AeNobo-G113A and AeNobo-F39L, were less inhibited by the flavonoids. Moreover, we found that DMG, which exhibits the strongest inhibitory activity against AeNobo in vitro in this study, shows larvicidal activity against *Ae. aegypti*. DMG is a more efficient larvicidal reagent than daidzein, which was identified as a larvicide for *Ae. aegypti* in a previous study [17]. To the best of our knowledge, this is the first study to identify a chemical compound that potentially inhibits a mosquito ecdysteroidogenic enzyme. Additionally, this study is the first to determine the crystal structure of a complex of insect GST and flavonoids.

We found that DMG inhibits the AeNobo enzymatic activity and exhibits larvicidal activity against *Ae. aegypti*, which might be due to the suppression of ecdysteroid biosynthesis. To date, several studies in mosquitoes have revealed that ecdysteroid signaling pathway regulates both mosquito abundance and competence, indicating that insecticides targeting any biological events related to ecdysteroids may be an asset for mosquito vector control [41]. Therefore, DMG is recognized as a potential IGR to target enzymes involved in the ecdysteroid biosynthesis pathway. However, since IGRs should specifically inhibit insect-specific biological processes, it is crucial to ensure that the IGRs do not impair any essential biological processes in organisms other than pests of interest. In this sense, the concern is that many flavonoids, including daidzein and luteolin, are well known to exert estrogen-like activities, as they can bind to estrogen receptors (ERs) and increase the proliferation of estrogen-sensitive cells [33–35]. However, our experiment using non-flavonoidal estrogenic compounds, including bisphenol A and diethylstilbestrol (Table 1, Additional file 2: Table S2), suggests that the estrogenic activity of compounds is not associated with the inhibition of AeNobo enzymatic activity. Moreover, DMG does not exhibit estrogenic activity because it does not promote ER binding activity to estrogen response elements of enhancer DNA region [33]. While the structure of DMG complexed with ERs has not been reported, the structure of genistein complexed with ERβ, deposited in Protein Data Bank (PDB ID:1X7J) [42, 43], gives us a hint for understanding why DMG does not exhibit the estrogenic activity. In the structure of ERβ-genistein complex, Ile373 of ERβ, which is close to the C5 of genistein, occupies the binding pocket beside the C5. Therefore, there might be a steric hindrance between a hydroxyl group at C6 of DMG and Ile373 of ERβ, although we remain cautious in our speculations because the ligand-binding pocket of ERs is known to show enough structural plasticity to adapt to their ligands [44]. Taken together, these results strongly indicate that there is no critical correlation between the estrogen-like activity of flavonoids and their inhibitory activity against AeNobo.

In this study, we showed that 2.5 ppm DMG treatment resulted in larval growth retardation and the reduced expression of the ecdysone-inducible gene *E74B*, suggesting an inhibitory effect of DMG on ecdysteroid biosynthesis in vivo. However, a 24-h treatment of DMG above 10 ppm evoked a high lethality, which appears to be different from the phenotype observed in loss of *nobo* function mutants of *D. melanogaster* and *B. mori*, in which molting is inhibited but organismal death does not occur immediately [23–25]. Therefore, further investigation is required to elucidate the specific inhibitory activity of DMG to AeNobo in vivo. Previous studies have identified DMG as an inhibitor of several enzymes in mammals, including phosphatidylinositol-3 kinase (PI3K) [45], protein kinase C α [46], cyclin-dependent kinase 1 and 2 [47], and the prolyl isomerase Pin1 [48]. In all cases, DMG directly binds to these proteins. Particularly, DMG inhibits PI3K activity via direct binding in an ATP-competitive manner [45]. By inhibiting these enzymes, DMG exhibits several beneficial effects on mammalian health, such as anti-cancer activity [47, 48], suppression of adipogenesis [45], suppression of osteoclast [49], antibacterial activity [50, 51], protective effect on cell death [52], and improvement of learning and memory [53]. Investigating how DMG binds to enzymes other than AeNobo and understanding the differences in binding modes between AeNobo and other enzymes at the molecular level would be beneficial.

The prevalence of resistance to mosquito pesticides that are currently being used worldwide, such as pyrethroid deltamethrin and temephos, are high in several areas [4]. Although some new strategies have been investigated for controlling the growing mosquito population [54, 55], the continuous need for the development of new insecticides targeting different molecules remains urgent. Our discovery of DMG as the most efficient AeNobo inhibitor provides a new strategy for the development of a novel environment-friendly IGR that controls mosquito population by inhibiting ecdysteroid biosynthesis. In the future, it would be intriguing to identify DMG-derivatives that inhibit AeNobo with a greater efficiency and to examine whether the DMG-derivatives exhibit higher larvicidal activities against *Ae. aegypti* than DMG. To further develop such highly active inhibitors, we plan to focus on a DMG-derivative that interacts with the aa of H-sites other than Glu-113 and Phe-39. In the crystal structures of AeNobo, some aa residues at the H-site, such as Ile-11, Met-117, Arg-118, and Ile-121, were found to be mobile in all subunits, indicating that these aa residues contribute only marginally to the interaction with DMG. Therefore, it should be interesting to design DMG-derivatives with hydrophobic functional groups that can interact with the H-site aa residues. Specifically, a DMG-derivative targeting Arg-118 might be a great seed compound for developing a mosquito-specific pesticide because Arg-118 is conserved among Nobo proteins from various mosquito species, whereas the 118th aa residue of Nobo proteins from other insects is serine or tyrosine [23, 30]. Therefore, our structural analysis will provide a basis for further consideration of DMG-based IGR development in the future.

## Conclusion

Here, we identified flavonoids, members of a polyphenolic class of secondary plant metabolites, as potential inhibitors of the ecdysteroidogenic regulator, Noppera-bo, in the yellow fever mosquito *Aedes aegypti*. Our biochemical and structure biological analyses revealed an essential mode of interaction between the flavonoids and the Noppera-bo protein underlying their inhibitory activity. Finally, we confirmed that DMG, the most efficient flavonoid for the inhibition of Noppera-bo, shows high larvicidal activity against *Ae. aegypti*.

## Methods

### Transgenic Drosophila melanogaster insects and genetics

*Drosophila melanogaster* flies were reared on standard agar-cornmeal medium at 25 °C under a 12 h:12 h light-dark cycle. The *nobo*^*KO*^ strain used has been previously described [23]. *phm-GAL4#22* (Research Resource Identifier (RRID):BDSC_80577) [56, 57] was kindly gifted by Michael B. O’Connor (University of Minnesota, USA).

The GAL4/UAS system [58] was used to overexpress the *AeNobo* gene in *D. melanogaster*. pUAST-attB plasmid [59], carrying the *AeNobo* coding sequence, was built using the custom gene synthesis service of VectorBuilder, Inc. Transformants were generated using the φC31 integrase system in the *P{CaryP}attP40* strain (RRID:BDSC_79604) [60]. The *w*^*+*^ transformants of pUAST-attB were established using standard protocols. Viability of *nobo*^*KO*^ animals, expressing the *nobo* transgene driven by *phm-GAL4#22*, was examined as described previously [23].

### Flavonoid chemicals

The following flavonoids were used in this study: biochanin A (>98%, Tokyo Chemical Industry, B4098), (+)-catechin hydrate (98%, Tokyo Chemical Industry, C0705), chrysin (98%, Alfa Aesar, L14178), cyanidin chloride (98%, Fujifilm Wako Chemicals, 030-21961), daidzein (DAI; ≥98%, Nagara Science, NH010102), desmethylglycitein (DMG; >95%, Tokyo Chemical Industry, T3473), *S*-equal (≥97%, Merck, SML2147), fisetin (>96%, Tokyo Chemical Industry, T0121), genistein (>98%, Tokyo Chemical Industry, G0272), 2′-hydroxyflavanone (>98%, Tokyo Chemical Industry, H1024), kaempferol (≥98%, Cayman Chemical Company, 11852), luteolin (95%, Fujifilm Wako Chemicals, 127-06241), myricetin (>97%, Tokyo Chemical Industry, M2131), naringenin (>98%, Alomone Labs, N-110), petunidin (≥98%, Cayman Chemical Company, 19755), quercetin (96.5%, Fujifilm Wako Chemicals, 512-58344), tamarixetin (>99%, Extrasynthese, 1140S), 5,3′,4′-trihydroxyflavone (>85%, Toronto Research Chemicals, T896685), and 7,3′,4′-trihydroxyflavone (>85%, Toronto Research Chemicals, T896780).

### Estrogenic compounds

The following estrogenic compounds were used in this study: 2-Amino-1-methyl-6-phenylimidazo[4,5-b]pyridine (≥98%, Sigma-Aldrich, 22590), 1-benzyl 2-butyl benzene-1,2-dicarboxylate (98%, eNovation, D584181), 16-benzylidene estrone (95%, OTAVA, 7569822), Biochanin A (>90%, InterBioScreen, BB_NC-02653), Biosphenol A (4,4′-propane-2,2-diyldiphenol) (90%, Vitas-M, STK801675), bis(2,4-dihydroxyphenyl)methanone (>90%, ChemBridge, 5222210), butyl 4-hydroxybenzoate (>90%, ChemDiv(LB), 0099-0145), 1,1-Dichloro-2,2-bis(4-chlorophenyl)ethene (90%, Sigma-Aldrich, 123897), diethylstilbestrol (4-[(*E*)-4-(4-hydroxyphenyl)hex-3-en-3-yl]phenol) (>98%, Tokyo Chemical Industry, D0526), Ferutinine (>90%, InterBioScreen, STOCK1N-32042), (*S*)-5-(4-hydroxy-3,5-dimethylphenyl)-1-methyl-2,3-dihydro-1H-inden-1-ol (90%, Sigma-Aldrich, SML1876), 4-[(1*S*,5*R*)-5-(hydroxymethyl)-8-methyl-3-oxabicyclo[3.3.1]non-7-en-2-yl]phenol (>90%, InterBioScreen, STOCK1N-10587), 4-[(1*R*,5*R*)-5-(hydroxymethyl)-6,8,9-trimethyl-3-oxabicyclo[3.3.1]non-7-en-2-yl]phenol (>90%, InterBioScreen, STOCK1N-13438), 4-[(1*R*,5*R*)-4,4,8-trimethyl-3-oxabicyclo[3.3.1]non-7-en-2-yl]phenol (>90%, InterBioScreen, STOCK1N-00708), propyl 4-hydroxybenzoate (>90%, ChemDiv(LB), 0099-0143), Resveratrol (>90%, InterBioScreen, BB_NC-02570), 4-[2,2,2-trichloro-1-(4-hydroxyphenyl)ethyl]phenol (90%, Vitas-M, STK996193), 4-(2,4,4-trimethylpentan-2-yl)phenol (>90%, Chemspace, PB425466960), α-zearalanol (>90%, InterBioScreen, STOCK1N-99337), and Zearalenone (>90%, InterBioScreen, STOCK1N-03962).

### Plasmid construction of Escherichia coli protein expression system

*Aedes aegypti* (SMK strain, 4th instar larvae, eight animals) cDNA was obtained by reverse transcription using ReverTra Ace qPCR RT Master Mix (Toyobo). *AeNobo* coding region was amplified from *Ae. aegypti* cDNA using PCR. The forward primer 5′-ATGTCCAAACCGGTGCTGTATTAC-3′ and the reverse primer 5′-CTATTTTTTCATTACAGCATGAAGTCTC-3′ were used for touchdown PCR. Touchdown PCR was conducted as follows: first, denaturation was performed for 10 s at 98 °C, followed by annealing for 30 s at 68 °C and extension for 1 min at 68 °C for 13 cycles. Next, denaturation was performed for 10 s at 98 °C, followed by annealing for 30 s at 55 °C and extension for 1 min at 68 °C for 30 cycles. The PCR product was ligated to pBluescript SK(-) to check and confirm the *AeNobo* sequence. After sequence verification, *AeNobo* was amplified from the vector through PCR using KOD-plus Neo polymerase (Toyobo) under the following conditions: pre-denaturation at 94 °C for 2 min, denaturation at 98 °C for 10 s, and extension at 68 °C for 30 s for 40 cycles. The product was extracted and ligated to pCold III (Takara Bio) for expression of the AeNobo-WT protein in *Escherichia coli*.

pCold III plasmids expressing the AeNobo-E113A and AeNobo-F39L were constructed using KOD-Plus-Mutagenesis Kit (Toyobo). The following primers were used: E113A-F (5′-CGTCGATCGTAATGCGAGGCTTGATC-3′) and E113A-R (5′-CTCTTTGGAACAGAACGGCATTGTTG-3′) for AeNobo-E113A and F39L-F (5′-AGAGAGAGAACATCTTTTGGAAG-3′) and F39L-R (5′-AAGAGGCGAACCAGTTTGAGTTC-3′) for AeNobo-F39L. We conducted inverse PCR using KOD-plus polymerase, pColdIII/AeNobo-WT plasmid, and the primer pair described above under the following conditions: pre-denaturation at 94 °C for 2 min, denaturation at 98 °C for 10 s, and extension at 68 °C for 6 min for 6 cycles. The PCR products were treated with *Dpn*I at 37 °C for 1 h, followed by self-ligation using T4 polynucleotide kinase and Ligation High. After the transformation of DH5α bacteria using the ligation products, we extracted plasmid DNA from the colonies and verified their sequences to confirm whether the appropriate point mutations were introduced.

### Protein expression and purification

Recombinant DmNobo protein was produced using an *E. coli* expression system as previously described [29, 30]. Similarly, recombinant AeNobo protein was produced using an *E. coli* expression system as follows: pCold III-AeNobo plasmid was transformed into the *E. coli* BL21 Star (DE3) strain (Thermo Fisher Scientific) for 30 min at 4 °C. The transformant was plated in Luria-Bertani (LB) medium supplemented with 100 μg/mL ampicillin and incubated at 37 °C overnight. Next, a bacterial colony from the plate was inoculated into 200 mL of LB supplemented with 100 μg/mL ampicillin (LB-amp medium) and shaken at 37 °C overnight for preculture. The preculture was transferred to 6 L of LB-amp medium for the main culture and shaken at 37 °C. After the optical density of the culture reached 1.0, Nobo protein expression was induced by incubation with 0.1 mM isopropyl β-D-1-thiogalactopyranoside at 15 °C overnight. Next, the bacterial cells were harvested via centrifugation at 4,000 × *g* for 15 min. The bacterial pellet was stored at −80 °C. The pellet from the 3-L culture was suspended in a lysis buffer (140 mM NaCl, 20 mM Tris-HCl at pH 8.0, and 1 mM dithiothreitol [DTT]). The cells were disrupted via sonication for 2 min at 70% amplitude with output 7 using the ULTRA5 HOMOGENIZER VP-305 (TAITEC) on ice. The soluble lysate was fractionated using centrifugation at 35,000 × *g* for 30 min. The supernatant was mixed with 10 mL of Glutathione Sepharose 4B beads (Cytiva) for 1 h at 4 °C for glutathione affinity purification. The beads were then collected and washed in lysis buffer. Proteins bound to the beads were eluted using 50 mL of elution buffer (10 mM GSH, 140 mM NaCl, 20 mM Tris-HCl at pH 8.0, and 1 mM DTT). Next, the eluent was concentrated to 5 mL and fractionated using size exclusion column chromatography with a HiLoad Superdex200 26/60 instrument (Cytiva) equilibrated using a size exclusion buffer (150 mM NaCl, 25 mM Tris-HCl at pH 8.0, and 5 mM DTT) at a flow rate of 1 mL/min. The purity of the fractions was evaluated using sodium dodecyl sulfate-polyacrylamide gel electrophoresis followed by Coomassie Brilliant Blue staining. Peak fractions were collected, and their buffer was replaced with another buffer (150 mM NaCl, 25 mM Tris-HCl at pH 8.0, 5 mM DTT, 10 mM GSH) by ultrafiltration conducted twice with an Amicon Ultra-15 30,000 MWCO instrument (Merck); proteins were then concentrated to 45 mg/mL. Protein concentration was measured by spectrophotometry using a NanoDrop ND-1000 spectrophotometer (Thermo Fisher Scientific) at an extinction coefficient (ε_280_) of 0.852 M^−1^ cm^−1^. Finally, the protein was stored at −80 °C.

### Measurement of specific activity of AeNobo protein in vitro

In vitro GST assays using 3,4-DNADCF were performed as described previously [29]. The stock solutions of AeNobo-WT, AeNobo-E113A, and AeNobo-F39L were 174.6, 227.8, and 291.4 ng/mL, respectively, in solution A (2 mM GSH, 100 mM sodium phosphate buffer at pH 6.5, 0.01% Tween20). Decreasing concentrations of AeNobo-WT, AeNobo-E113A, and AeNobo-F39L, ranging from 174.6 to 3.0 ng/mL, from 227.8 to 4.0 ng/mL, and from 291.4 to 5.0 ng/mL, respectively, were prepared by 2/3-fold serial dilution with solution A. The AeNobo dilution series was mixed with an equal volume of solution B (100 mM sodium phosphate buffer at pH 6.5, with 2mM 3,4-DNADCF in 0.2% dimethyl sulfoxide (DMSO) as a co-solvent) in each well of a 96-well plate to initiate the catalytic reaction of DmNobo. In these wells, the final concentrations of AeNobo-WT, AeNobo-E113A, and AeNobo-F39L ranged from 87.3 to 1.5 ng/mL, from 113.9 to 2.0 ng/mL, and from 145.7 to from 2.5 ng/mL, respectively. The glutathione-conjugated product was excited at 485 nm wavelength, and the fluorescence intensity at 535 nm or 538 nm wavelength (*F*_*measured*_) was measured every 30 s for 20 min using an Infinite 200 PRO instrument (Tecan) or Fluoroskan Ascent™ FL (Thermo Fisher Scientific). The specific activity of AeNobo enzymes was determined as previously described [30].

### GST activity inhibition assay

The IC_50_ value was measured using an in vitro assay system as described previously [29]. A dilution series of compounds, ranging from 2.5 mM to 0.127 μM for DMG and from 2.5 mM to 4.9 μM for other compounds, was prepared by 2-fold serial dilution in DMSO. Five microliters of each diluted compound solution was mixed with 245 μL of solution A (100 mM sodium phosphate buffer at pH 6.5, 0.01% Tween 20, 2 mM GSH, and 50 ng/mL AeNobo-WT, 100 ng/mL AeNobo-E113A, and 200 ng/mL F39L). One hundred microliters of the mixture was dispensed into wells of a 96-well plate. One hundred microliters of solution B (0.2 μM 3,4-DNADCF and 100 mM sodium phosphate buffer at pH 6.5) was added to each well. In summary, the final reaction system comprised 100 mM sodium phosphate buffer (pH 6.5), 1 mM GSH, 0.005% Tween 20, 0.1 μM 3,4-DNACDF, and 25 ng/mL AeNobo, 50 ng/mL AeNobo-E113A, or 100 ng/mL AeNobo-F39L protein. The fluorescence intensity derived from 4-GS-3-NADCF, a product of this reaction, was measured for 3 min. IC_50_ values were estimated as described previously [30]. The enzymatic assays under each condition were performed at least twice independently.

### High-throughput screening of 9600 compounds

To identify inhibitors of AeNobo enzymatic activity, a high-throughput screening was performed as described in a previous study [29]. Briefly, a core library of 9600 compounds obtained from the Drug Discovery Initiative, The University of Tokyo, was utilized for screening. In this screen, the enzymatic activity of AeNobo was detected using 3,4-DNADCF [29]. First, 1 μL of solution A (11.2 ng/mL AeNobo protein, 100 mM sodium phosphate at pH 6.5, 2 mM GSH, and 0.005% Tween 20) was dispensed into each well of a 1536-well plate (1536 Black SV/NB/FI, #784900, Greiner Bio-One) together with 0.01 μL of compounds at a concentration of 2 mM. Then, 1 μL of solution B (4 μM 3,4-DNADCF, 100 mM sodium phosphate at pH 6.5, and 0.005% Tween 20) was added into each well. In summary, the reaction system comprised 5.6 ng/mL AeNobo protein, 2 μM 3,4-DNADCF, 1 mM GSH, 0.005% Tween 20, and 100 mM sodium phosphate at pH 6.5, together with each compound at 10 μM. The plate was incubated for 30 min at 25–27 °C and 2 μL of 10 mM N-ethylmaleimide was added into each well to stop the reaction. For the first screen, 90 compounds that inhibited the enzymatic activity of AeNobo by more than 50% were selected. For the second screen, we assayed the IC_50_ values of the compounds against the enzymatic activity of AeNobo. Compounds with IC_50_ values lower than 10 μM were defined as hit compounds.

### Crystallization

A 100 mM GSH stock solution was prepared in a buffer composed of 150 mM NaCl, 25 mM Tris-HCl at pH 8.0, and 5 mM DTT. The GSH stock solution was diluted to 30 mM GSH by adding an AeNobo protein solution for co-crystallization. The AeNobo protein solution was centrifuged at 13,500 × *g* for 30 min to remove protein aggregates.

An initial crystallization assay was performed using a Protein Crystallization System (PXS) [61] following the sitting drop vapor diffusion crystallization method with 0.2 μL of each protein solution and one of the reservoir solutions from the following kits: Crystal Screen 1 & 2 (Hampton Research, Aliso Viejo, CA, USA), Index (Hampton Research), PEGIon (Hampton Research), PEGIon2 (Hampton Research), Wizard I & II (Molecular Dimensions, Suffolk, UK), PEGs II Suite (Qiagen), Protein Complex Suite (Qiagen), Stura FootPrint Screen (Molecular Dimensions), and MembFac (Hampton Research) at 20 °C. Under these conditions, crystals formed only when the reservoir solution was composed of 30% (w/v) PEG 4000, 0.1 M Tris-HCl at pH 8.5, and 0.2 M magnesium chloride. The conditions were optimized using the hanging drop vapor diffusion method, through which crystals formed in drops of 1 μL, each containing 45 mg/mL AeNobo protein and a reservoir solution (32.5% (w/v) PEG 4000, 0.1 M Tris-HCl, pH 7.5, 0.5 M calcium chloride) at 20 °C. To obtain a structure complexed with flavonoids, AeNobo crystals were soaked in 30 mM luteolin or DMG suspensions in the reservoir solution for 1 day at 20 °C. As luteolin did not completely dissolve in the reservoir solution at 30 mM, AeNobo crystals were soaked in a reservoir solution saturated with luteolin for 1 day at 20 °C.

### X-ray crystallography

Crystals were soaked in a cryoprotectant solution (30% (w/v) Polyvinylpyrrolidone K 15 Average Molecular Wt. 10000 (Tokyo Chemical Industry, P0471)/reservoir solution), picked with cryo-loops (MiTeGen), flash frozen in liquid nitrogen, and packed in Uni-pucks (Molecular Dimensions). X-ray diffraction experiments for crystals of AeNobo-GSH, AeNobo-GSH-daidzein, AeNobo-GSH-luteolin, and AeNobo-GSH-DMG complexes were performed at beamlines BL-17A, BL-5A, NE-3A, and BL-5A, respectively, at the Photon Factory, High Energy Accelerator Research Organization (KEK), Tsukuba, Japan. The collected datasets were processed and scaled using XDS (RRID:SCR_015652) [62] and AIMLESS (RRID:SCR_015747) [63], respectively. Space groups were determined using POINTLESS (RRID:SCR_014218) [64]. Phases for AeNobo-GSH were calculated using the molecular replacement method with the DmNobo structure (PDB ID: 6KEM) [37] as a template, and those for AeNobo-GSH-daidzein, AeNobo-GSH-luteolin, and AeNobo-GSH-DMG were calculated using the AeNobo-GSH structure. Model building and crystallographic refinement were performed by COOT (RRID:SCR_014222) and PHENIX.REFINE (RRID:SCR_016736) [65, 66]. The crystallographic statistics are summarized in Additional file 2: Table S3.

### MD simulation

The structures of AeNobo-GSH-daidzein and AeNobo-GSH-DMG were processed to assign bond orders and hydrogenation. The ionization states of each compound and GSH at pH 7.0 ± 2.0 were predicted using Epik [67], and H-bond optimization was conducted using PROPKA [68]. Energy minimization was performed in Maestro (Schrödinger) using the OPLS3e force field [69]. Each E113A mutation model for MD simulation was constructed in Maestro and treated using the same protocol. MD simulations were prepared using the Molecular Dynamics System Setup Module of Maestro. All structures were subjected to energy minimization and placed in an orthorhombic box with a buffer distance of 10 Å to create a hydration model, and the SPC water model [70] was used for the hydration model. NaCl (0.15 M) served as the counter ion to neutralize the system. The MD simulations were performed using the Desmond software, version 2.3 (Schrödinger) (RRID:SCR_014575). The cutoff radii for van der Waals and the time step, initial temperature, and pressure of the system were set to 9 Å, 2.0 femtoseconds, 300 K, and 1.01325 bar, respectively. The sampling interval during the simulation was set to 100 ps. Finally, we performed MD simulations using the NPT ensemble for 1 μs. All trajectories from MD simulations were aligned to the initial structure with protein Cα, and ligand RMSD values were calculated based on ligand heavy atoms.

### Mosquito rearing

The *Ae. aegypti* strain used in this study, which originated from the Liverpool strain, was a gift from Ryuichiro Maeda (Obihiro University of Agriculture and Veterinary Medicine). Five hundred pupae were harvested in a plastic cup and placed within a nylon mesh cage (bottom 27 cm × 27 cm, top 25 cm × 25 cm, height 27 cm). A 50-mL glass flask, inserted with a filter paper (#1001-125, Whatman), containing 10% sucrose solution was placed in the nylon mesh cage. The cage rearing the emerged adults was kept in an incubator (MIR-254-PJ, Panasonic Co.) set at 27 °C with humidity over 90% in a standard 12 h:12 h light-dark cycle. The sucrose solution was changed every 3–4 days. Adult females (at 7–14 days after eclosion) were blood fed and allowed to lay eggs on a wet filter paper, 3 to 4 days after engorgement. Eggs laid on the filter paper were washed once with RO water and kept in a plastic container with wet paper for further egg maturation. After a week, the lid of the container was left slightly open to slowly dry the eggs for storage.

### Aedes aegypti larvicidal assay

The dried eggs on filter papers were soaked in distilled water. Three hours after soaking, the first instar larvae were transferred to 30 mL of fresh distilled water in a 50-mL plastic cup with a lid containing air holes. In the rearing water, 2 mg of the powdered goldfish food (Hikari Medium Grain, Kyorin Co., Ltd.) was added to each cup as food for *Ae. aegypti* larvae. In each cup, we poured the 30 mL rearing water containing 1 ppm, 10 ppm, or 100 ppm of the compounds with 0.1% ethanol at the final concentration, followed by placing 20 larvae. Twenty-four hours after the addition of flavonoids, the number of living and dead larvae was recorded. Larvacidal assays under each condition were performed 5 times independently. The larval instars 24 h after the addition of DMG were identified by measuring the transverse diameter of the *Ae. aegypti* larval head [39]. Photographs of whole bodies of larvae were taken and the head diameter was then measured using ImageJ software [71].

### Reverse transcription-quantitative PCR (RT-qPCR)

Preparation of control and DMG-treated larvae was conducted as described above in the previous section “*Aedes aegypti* larvicidal assay.” Twenty-four hours after the addition of flavonoids, 20-30 *Ae. aegypti* larvae for each sample were homogenized in RNAiso Plus (Takara Bio Inc.) to extract total RNA. RNA was reverse transcribed to synthesize cDNA using ReverTra Ace qPCR RT Master Mix with gDNA Remover (Toyobo). cDNA samples were used as templates for qPCR using THUNDERBIRD SYBR qPCR Mix (Toyobo) on a Thermal Cycler Dice Real Time System (Takara Bio Inc.). The mRNA level of *E74B* was normalized to that of an endogenous control *ribosomal protein 49* gene (*rp49*), and the relative fold change was calculated. The normalized *E74B* expression level was compared using the ΔΔCt method. The primers for *E74B* were AeaegE74B-Fwd (5′-GCCTTGGAATTCCACTCACAAA-3′) and AeaegE74B-Rev (5′-GGTCTGGTGAACGGACTACACC-3′). The primers for *rp49* were Aeaeg-rp49-Fwd (5′-TCGGCAGTCTTGCCAACCCTGA-3′) and Aeaeg-rp49-Rev (5′-AGCTTATCATACCGACGTTCCGAA-3′).

### Drosophila melanogaster survival assay

A 0.1% (w/v) DMG stock solution was prepared in 100% DMSO. Ten microliters of the DMG stock solution or 10 μL of DMSO alone was mixed with 10 g of standard cornmeal medium and 1 mL of autoclaved water. The volume of the mixed food was approximately 10 mL, and therefore, the food contained 0.1% DMSO with or without 10 ppm DMG. Two grams of the food were dispensed into each 12-mL plastic vial (Sarstedt). *D. melanogaster* wild-type Canton-S were given the opportunity to lay eggs on a grape juice agar with yeast paste for 12 h. After egg collection, the embryos were reared at 25 °C for 24 h, and then 20 1st instar larvae were transferred to each vial and reared at 25°C. The numbers of pupae were counted daily, and the timing of pupation was recorded.

## Supplementary Information

**Additional file 1 : Figure S1.** Glu-113 of AeNobo and Asp-113 of DmNobo. (*A*) Comparison of predicted amino acid sequences between *Ae. aegypti* LOC5569853 proteins encoded by the *LOC5569853* gene and the wildtype DmNobo proteins. There are two predicted amino acid sequences in the GenBank database, EAT40301.1 and XM_001658698.3, whose amino acid (aa) lengths are 220 and 271, respectively. The 220-aa protein, rather than the 271-aa protein, is substantially similar to DmNobo. Therefore, we used the gene encoding the 220-aa protein for the transgenic rescue experiment (Additional file 2: Table S1) and all biochemical and structure biological analyses in this study. Glu-113 of AeNobo and Asp-113 of DmNobo are marked with a red box. (*B*) The hydrogen bonds between Asp-113 of DmNobo and 17β-estradiol, Glu-113 of AeNobo and luteolin, and Glu-113 of AeNobo and daidzein. Carbon atoms of 17β-estradiol and flavonoids are colored pink and green, respectively. Oxygen and nitrogen atoms are colored red and blue, respectively. Hydrogen bonds are illustrated by dashed yellow lines. As shown in the most right panel (Superposed), Asp-113 of DmNobo and Glu-113 of AeNobo are present at the similar location. **Figure S2.** 17β-estradiol inhibitory activity against AeNobo is less than that against DmNobo. (*A*) Chemical structure of 17β-estradiol. (*B*) Inhibition of the GSH conjugation activities of DmNobo (left) and AeNobo (right) determined using an artificial fluorescent substrate 3,4-DNADCF in the presence of 17β-estradiol. Each relative activity is defined as the ratio of activity compared between the respective proteins without 17β-estradiol. All the data points in triplicate (for DmNobo) or duplicate (for AeNobo) assays are indicated. **Figure S3**. Chemical structure of 14 flavonoids used in our initial chemical screen. 2′-hydroxyflavanone was used for the experiment shown in Fig. 1A. Thirteen other flavonoids, including the subclasses of flavonone, flavone, isoflavone, flavonol, isoflavan, and anthocyanidin, were used for the experiment shown in Fig. 1B. The IC_50_ values of the 14 flavonoids are shown in Table 1. **Figure S4**. Purification of AeNobo-WT. (*A*) AeNobo-WT was purified using size exclusion column chromatography. The UV charts of AeNobo-WT and AeNobo-E113A are shown using blue and orange lines, respectively. The peak points of marker proteins are indicated using black solid lines. The marker proteins used were: β-amylase from sweet potato (223.8 kDa; a), alcohol dehydrogenase from *Saccharomyces cerevisiae* (146.8 kDa; b), bovine serum albumin (66.5 kDa; c), human GSTP1-1 (46.7 kDa in solution; d), and RNase A (13.7 kDa; e). (*B*) The asymmetric unit of AeNobo is composed of four chains: A, B, C, and D. (*C*) Biological dimers with a symmetry-related subunit by a crystallographic two-fold axis: Chain A–Chain A´, Chain B–Chain B´´, Chain C–Chain D´´´, and Chain D–Chain C´´´´. (*D*) Overlaid structures of the 4 dimer pairs shown in *C*. **Figure S5**. Simulated annealing (SA) omit maps for GSH and flavonoids in all chains of AeNobo. *mF*_*o*_*-DF*_*c*_ maps (green) (3.0 σ) are shown. Carbon atoms of AeNobo and GSH and flavonoids are colored deep teal and wheat, respectively. Carbon atoms of daidzein (*B*), luteolin (*C*), and desmethylglycitein (*D*) are colored orange, light violet, and gray, respectively. Oxygen, nitrogen, and sulfur atoms are colored red, blue, and yellow, respectively. **Figure S6.** Interaction between AeNobo and GSH. Carbon atoms of GHS, daidzein, luteolin, and desmethylglycitein (DMG) are colored wheat, orange, light violet, and gray, respectively. Oxygen, nitrogen, and sulfur atoms are colored red, blue, and yellow, respectively. Water molecules are shown as yellow spheres. Hydrogen bonds are illustrated by yellow dashed line. (*A*) Position of GSH and surrounding amino acids of the chain D of AeNobo complexed with GSH (left), with GSH and daidzein (middle), and with GSH and desmethylglycitein (DMG). (*B*) Position of GSH and surrounding amino acids of the chain B of AeNobo complexed with GSH (left), and with GSH and luteolin. **Figure S7.** Hydrogen bonds between all chains of AeNobo and daidzein, luteolin, and desmethylglycitein (DMG). Hydrogen bonds are illustrated by yellow dashed line. (*A*) Position of Glu-113 of AeNobo-GSH with (orange) or without (pink) daidzein. (*B*) Position of Glu-113 of AeNobo-GSH with (red) or without (pink) luteolin. (*C*) Position of Glu-113 of AeNobo-GSH with (gray) or without (pink) DMG. Note that the hydrogen bonds are observed in all 4 chains. **Figure S8**. Distribution of RMSD values of daidzein and desmethylglycitein (DMG) in AeNobo-WT and AeNobo-E113A. All RMSD values were calculated from three independent MD simulations for each complex (3 × 10000 snapshots). ** *p*<0.01 Student’s *t*-test. **Figure S9**. Phe-39 of AeNobo. (*A, B*) A 3-dimentional position of desmethylglycitein (DMG) (*A*) and luteolin (*B*) with Glu-113 and Phe-39 of AeNobo. Carbon atoms of DMG and luteolin are colored gray and light violet, respectively. Oxygen, nitrogen, and sulfur atoms are colored red, blue, and yellow, respectively. (*A*) The hydrogen bonds (dashed yellow lines) between Glu-113 of AeNobo and DMG. Additionally, DMG forms a CH-π interaction (dashed black line) with Phe-39. (*B*) The hydrogen bonds (dashed yellow lines) between Glu-113 of AeNobo and luteolin. There is no obvious interaction between Phe-39 and luteolin. (*C, D*) Inhibition of the GSH conjugation activities of AeNobo-WT (blue) and AeNobo-F39L (red), determined using an artificial fluorescent substrate 3,4-DNADCF in the presence of DMG (*C*) or luteolin (*D*). Each relative activity is defined as the ratio of activity compared between the respective proteins without flavonoids. All the data points in triplicate (for DmNobo) and duplicate (for AeNobo) assays are indicated. **Figure S10**. Pupariation ratio of control and 10 ppm DMG-treated *D. melanogaster.* The pupariation timing was not changed in 10 ppm DMG-treated larvae (red, *n*=240) as compared with control larvae (grey, *n*=240).

**Additional file 2 : Table S1.** Viability of *nobo*^*KO*^ animals expressing AeNobo. **Table S2**. Inhibitory activity of estrogenic compounds against AeNobo. **Table S3.** Crystallographic statistics. **Table S4.** RMSD (Å) of Cα atoms of the chains A/B/C/D. **Table S5**. Inhibitory activity of luteolin derivatives against AeNobo. **Table S6**. The head diameter of control and DMG-treated larvae.

**Additional file 3 : Movie 1.** A representative trajectory of MD simulations of AeNobo-WT, GSH, and daidzein. Simulation time: 1,000 ns; Sampling Interval: 100 ps. AeNoBo-WT is represented by the cartoon model, while E113 and daidzein are represented by the stick model. Daidzein is imposed at the middle-right in the AeNobo-WT protein. All waters and ions were omitted for clarification purposes.

**Additional file 4 **: **Movie 2**. A representative trajectory of MD simulations of AeNobo-E113A, GSH, and daidzein. Simulation time: 1,000 ns; Sampling Interval: 100 ps. AeNoBo-E113A is represented by the cartoon model, while A113 and daidzein are represented by the stick model. Daidzein is imposed at the middle-right in the AeNobo-E113A protein. All waters and ions were omitted for clarification purposes.

**Additional file 5 **: **Movie 3.** A representative trajectory of MD simulations of AeNobo-WT, GSH, and desmethylglycitein. Simulation time: 1,000 ns; Sampling Interval: 100 ps. AeNoBo-WT is represented by the cartoon model, while E113 and Desmethylglycitein are represented by the stick model. Desmethylglycitein is imposed at the middle-right in the AeNobo-WT protein. All waters and ions were omitted for clarification purposes.

**Additional file 6 **: **Movie 4.** A representative trajectory of MD simulations of AeNobo-E113A, GSH, and desmethylglycitein. Simulation time: 1,000 ns; Sampling Interval: 100 ps. AeNoBo-E113A is represented by the cartoon model, while A113 and Desmethylglycitein are represented by the stick model. Desmethylglycitein is imposed at the middle-right in the AeNobo-E113A protein. All waters and ions were omitted for clarification purposes.

## Data Availability

The X-ray data and coordinates presented in this paper are deposited in the Protein Data Bank (https://pdbj.org/), under the following PDB IDs: 7EBT, 7EBU, 7EBV, and 7EBW. Other datasets used and/or analyzed during the current study are available from the corresponding author on reasonable request.

## Abbreviations

AeNobo: *Aedes aegypti* Noppera-bo
DMG: Desmethylglycitein
DmNobo: *Drosophila melanogaster* Noppera-bo
DMSO: Dimethyl sulfoxide
3,4-DNADCF: 3,4-Dinitrobenzamidedichlorofluorescein
ER: Estrogen receptor
GSH: Glutathione
GST: Glutathione *S*-transferase
IC_50_: Concentration of 50% inhibition
IGR: Insect growth regulator
LD_50_: 50% lethal dose
MD: Molecular dynamics
Nobo: Noppera-bo
PI3K: Phosphatidylinositol-3 kinase
qPCR: Quantitative polymerase chain reaction
RMSD: Root mean square deviation
RT: Reverse transcription
WT: Wild-type
