# Supplementary material for: Molecular action of larvicidal flavonoids on ecdysteroidogenic glutathione S-transferase Noppera-bo in Aedes aegypti
Source: BMC Biol. 2022 Feb 17;20:43. doi: 10.1186/s12915-022-01233-2 (PMC8851771; doi:10.1186/s12915-022-01233-2)
Supplement: Supplementary file 1 — Additional file 1 : Figure S1. Glu-113 of AeNobo and Asp-113 of DmNobo. (A) Comparison of predicted amino acid sequences between Ae. aegypti LOC5569853 proteins encoded by the LOC5569853 gene and the wildtype DmNobo proteins. There are two predicted amino acid sequences in the GenBank database, EAT40301.1 and XM_001658698.3, whose amino acid (aa) lengths are 220 and 271, respectively. The 220-aa protein, rather than the 271-aa protein, is substantially similar to DmNobo. Therefore, we used the gene encoding the 220-aa protein for the transgenic rescue experiment (Additional file 2: Table S1) and all biochemical and structure biological analyses in this study. Glu-113 of AeNobo and Asp-113 of DmNobo are marked with a red box. (B) The hydrogen bonds between Asp-113 of DmNobo and 17β-estradiol, Glu-113 of AeNobo and luteolin, and Glu-113 of AeNobo and daidzein. Carbon atoms of 17β-estradiol and flavonoids are colored pink and green, respectively. Oxygen and nitrogen atoms are colored red and blue, respectively. Hydrogen bonds are illustrated by dashed yellow lines. As shown in the most right panel (Superposed), Asp-113 of DmNobo and Glu-113 of AeNobo are present at the similar location. Figure S2. 17β-estradiol inhibitory activity against AeNobo is less than that against DmNobo. (A) Chemical structure of 17β-estradiol. (B) Inhibition of the GSH conjugation activities of DmNobo (left) and AeNobo (right) determined using an artificial fluorescent substrate 3,4-DNADCF in the presence of 17β-estradiol. Each relative activity is defined as the ratio of activity compared between the respective proteins without 17β-estradiol. All the data points in triplicate (for DmNobo) or duplicate (for AeNobo) assays are indicated. Figure S3. Chemical structure of 14 flavonoids used in our initial chemical screen. 2′-hydroxyflavanone was used for the experiment shown in Fig. 1A. Thirteen other flavonoids, including the subclasses of flavonone, flavone, isoflavone, flavonol, isoflavan, and [file 12915_2022_1233_MOESM1_ESM.pdf]

Fig. S1

A

EAT40301.1	-	-	-	-	-	-	-	-	-	-	-	-	-	-	-	-	-	-	-	-	-	-	-	-	-	-	-	-	-	-
XM_001658698.3	M	L	I	S	C	S	V	R	S	T	H	F	V	R	A	I	S	Y	R	D	Y	K	I	S	I	L	E	L	V	W
DmNobo_WT	-	-	-	-	-	-	-	-	-	-	-	-	-	-	-	-	-	-	-	-	-	-	-	-	-	-	-	-	-	-

EAT40301.1	-	-	-	-	-	-	-	-	-	-	-	-	-	-	-	-	-	-	-	-	-	M	S	K	P	V	L	Y	Y	D
XM_001658698.3	I	V	F	N	F	C	F	S	F	L	F	L	F	G	F	C	L	E	D	L	T	M	S	K	P	V	L	Y	Y	D
DmNobo_WT	-	-	-	-	-	-	-	-	-	-	-	-	-	-	-	-	-	-	-	M	S	Q	P	K	P	I	L	Y	Y	D

EAT40301.1	D	I	S	P	P	V	R	G	V	L	L	T	V	A	A	L	G	I	K	D	Q	V	E	L	K	L	V	R	L	F
XM_001658698.3	D	I	S	P	P		R	G	V	L	L	T	V	A	A	L	G	I	K	D	Q	V	E	L	K	L	V	R	L	F
DmNobo_WT	E	R	S	P	P	V	R	S	C	L	M	L	I	K	L	L	D	I	D	-	-	V	E	L	R	F	V	N	L	F

EAT40301.1	E	R	E	H	L	L	E	D	F	V	K	L	N	P	L	H	A	V	P	V	L	K	H	D	D	L	V	L	T	D
XM_001658698.3	E	R	E	H	L	L	E	D	F	V	K	L	N	P	L	H	A	V	P	V	L	K	H	D	D	L	V	L	T	D
DmNobo_WT	K	G	E	Q	F	Q	K	D	F	L	A	L	N	P	Q	H	S	V	P	T	L	V	H	G	D	L	V	L	T	D

EAT40301.1	S	H	A	I	I	M	Y	L	C	D	I	F	G	Q	D	G	D	F	S	L	K	D	P	K	Q	R	A	R	V	H
XM_001658698.3	S	H	A	I	I	M	Y	L	C	D	I	F	G	Q	D	G	D	F	S	L	K	D	P	K	Q	R	A	R	V	H
DmNobo_WT	S	H	A	I	L	I	H	L	A	E	K	F	D	E	G	G	S	L	W	P	Q	E	H	A	E	R	M	K	V	L

														113																
EAT40301.1	N	R	L	C	F	N	N	A	V	L	F	Q	R	E	S	I	V	M	R	G	L	I	N	R	S	I	V	T	L	E
XM_001658698.3	N	R	L	C	F	N	N	A	V	L	F	Q	R	E	S	I	V	M	R	G	L	I	N	R	S	I	V	T	L	E
DmNobo_WT	N	L	L	L	F	E	C	S	F	L	F	R	R	D	S	D	F	M	S	A	I	V	R	Q	G	F	A	N	V	D

EAT40301.1	D	-	H	H	L	K	P	V	Q	E	A	Y	D	C	L	E	V	Y	L	T	N	S	K	F	V	A	C	D	Q	L
XM_001658698.3	D	-	H	H	L	K	P	V	Q	E	A	Y	D	C	L	E	V	Y	L	T	N	S	K	F	V	A	C	D	Q	L
DmNobo_WT	V	A	H	H	E	R	K	L	T	E	A	Y	I	I	M	E	R	Y	L	E	N	S	D	F	M	A	G	P	Q	L

EAT40301.1	T	V	A	D	F	P	I	V	A	C	M	S	T	V	G	M	V	C	P	L	S	T	S	R	W	P	K	T	A	A
XM_001658698.3	T	V	A	D	F	P	I	V	A	C	M	S	T	V	G	M	V	C	P	L	S	T	S	R	W	P	K	T	A	A
DmNobo_WT	T	L	A	D	L	S	I	V	T	T	L	S	T	V	N	L	M	F	P	L	S	-	-	Q	F	P	R	L	R	R

EAT40301.1	W	F	E	T	M	K	Q	L	P	Y	Y	Q	Q	A	N	Q	V	G	V	D	K	L	K	E	R	L	H	A	V	M
XM_001658698.3	W	F	E	T	M	K	Q	L	P	Y	Y	Q	Q	A	N	Q	V	G	V	D	K	L	K	E	R	L	H	A	V	M
DmNobo_WT	W	F	T	A	M	Q	Q	L	D	A	Y	-	E	A	N	C	S	G	L	E	K	L	R	Q	T	M	E	S	V	G

EAT40301.1	K	K	-	-	-	-	-	-	-	-	-	-	-	-	-	-	220aa
XM_001658698.3	K	K	-	-	-	-	-	-	-	-	-	-	-	-	-	-	271aa
DmNobo_WT	S	F	Q	F	P	S	S	S	A	V	V	T	E	K	V	E	232aa

B

D113 E113 E113 D113/E113

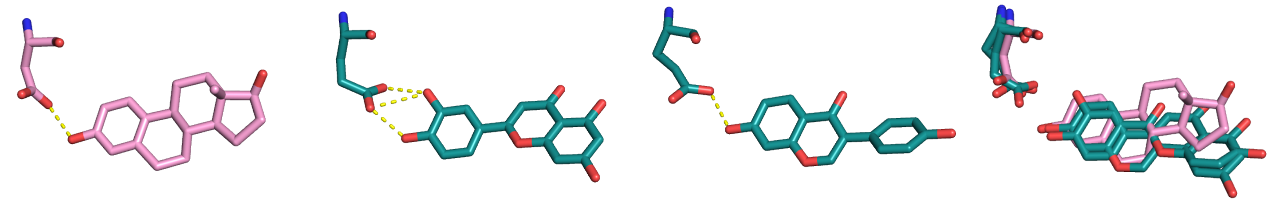

DmNobo-17 β estradiol AeNobo-Luteolin AeNobo-Daidzein Superposed

Fig. S2

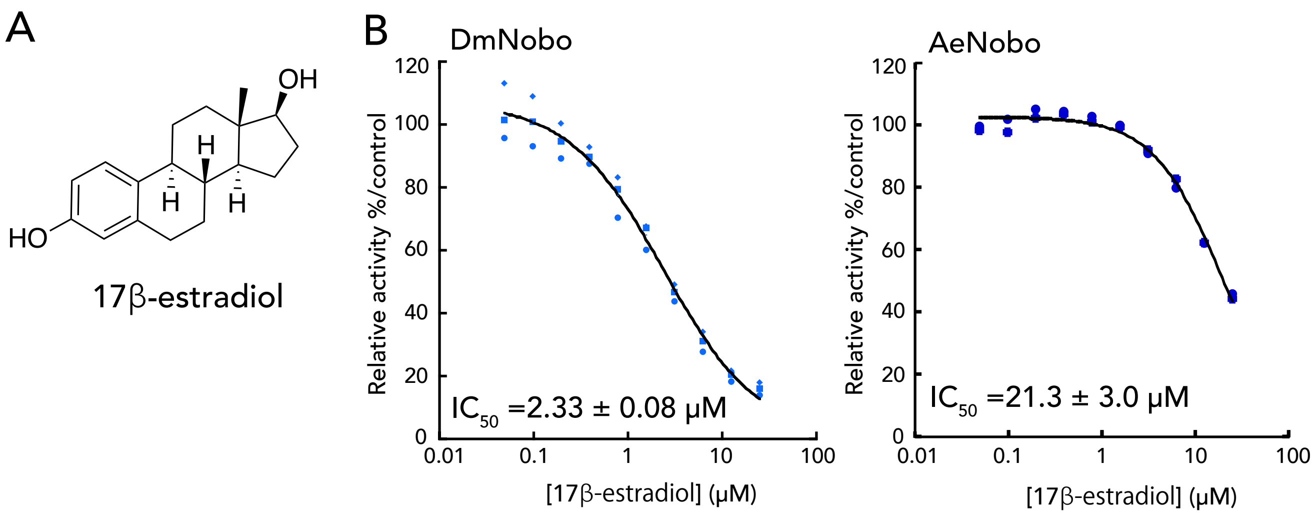

A
OH
H
H
H
HO
17β-estradiol
B
DmNobo
120
100
80
60
40
20
0
Relative activity %/control
IC50 =2.33 ± 0.08 μM
0.01
0.1
1
10
100
[17β-estradiol] (μM)
AeNobo
120
100
80
60
40
20
0
Relative activity %/control
IC50 =21.3 ± 3.0 μM
0.01
0.1
1
10
100
[17β-estradiol] (μM)

Fig. S3

Subclass	Chamical structure
Flavanone	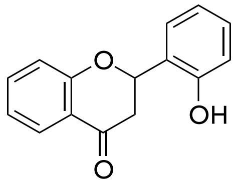 2′-hydroxyflavanone 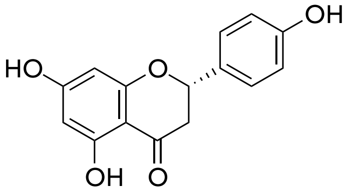 Naringenin
Flavone	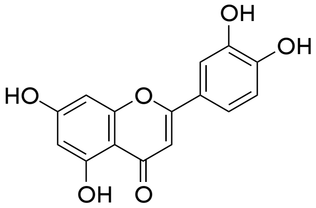 Luteolin
Isoflavone	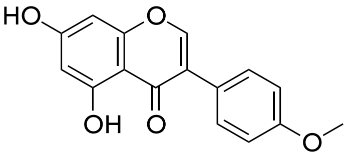 Biochanin A 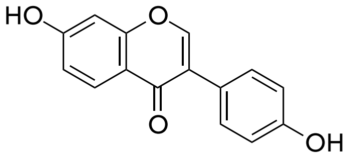 Daidzein
Flavonol	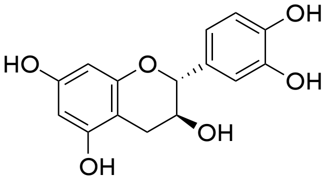 (+)Catechin hydrate 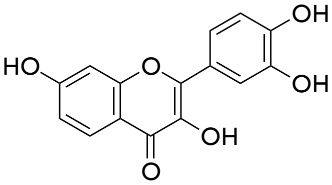 Fisetin 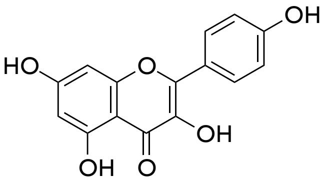 Kaempferol 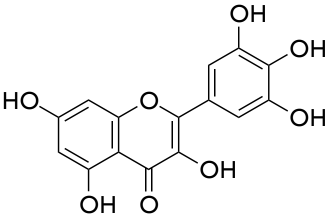 Myricetin 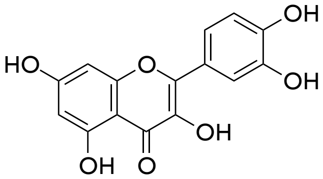 Quercetin 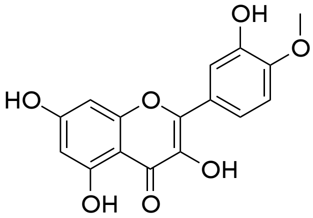 Tamarixetin
Isoflavan	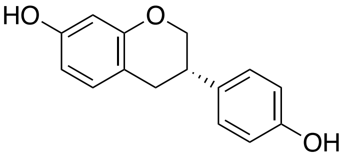 *S*-equol
Anthocyanidin	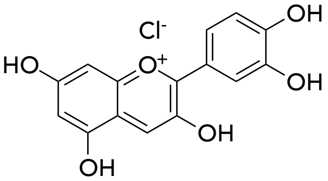 Cyanidin chloride 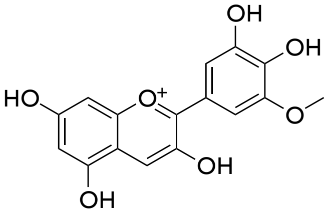 Petunidin

Fig. S4

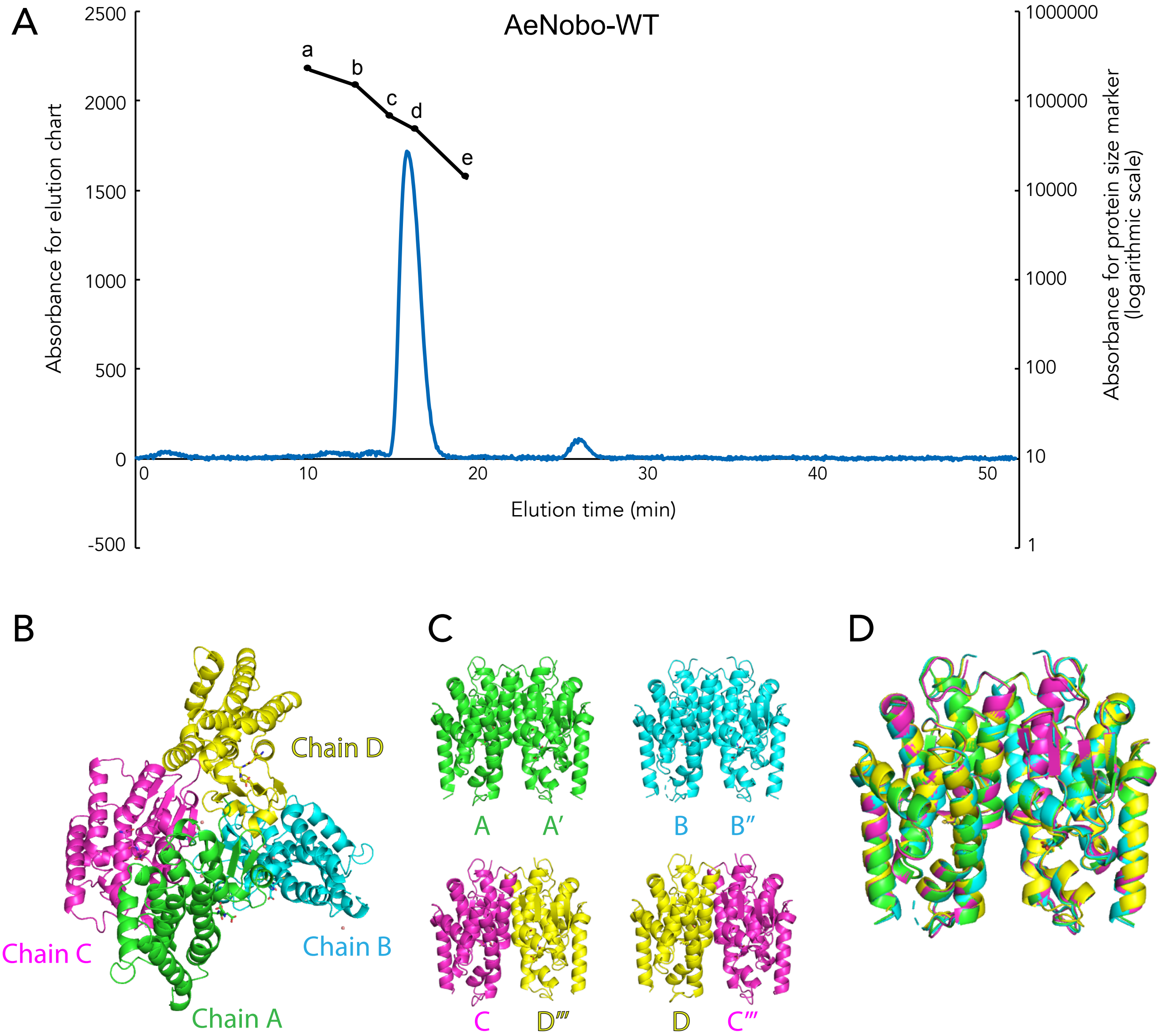
A
AeNobo-WT
Absorbance for elution chart
Absorbance for protein size marker (logarithmic scale)
Elution time (min)
a
b
c
d
e
2500
2000
1500
1000
500
0
-500
1000000
100000
10000
1000
100
10
1
0
10
20
30
40
50
B
Chain D
Chain C
Chain B
Chain A
C
A
A′
B
B″
C
D‴
D
C‴
D

Fig. S5

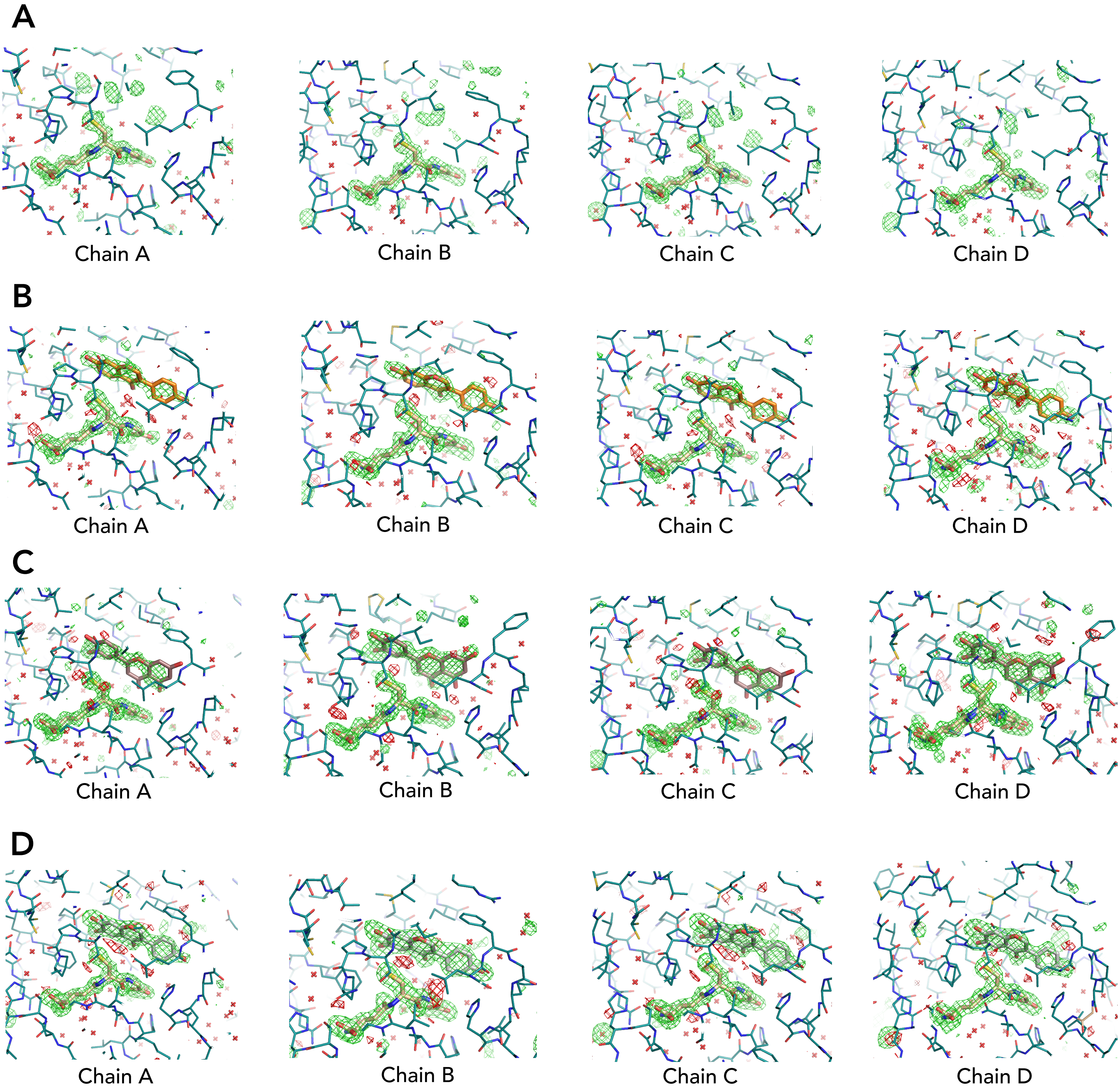
A
Chain A
Chain B
Chain C
Chain D
B
Chain A
Chain B
Chain C
Chain D
C
Chain A
Chain B
Chain C
Chain D
D
Chain A
Chain B
Chain C
Chain D

Fig. S6

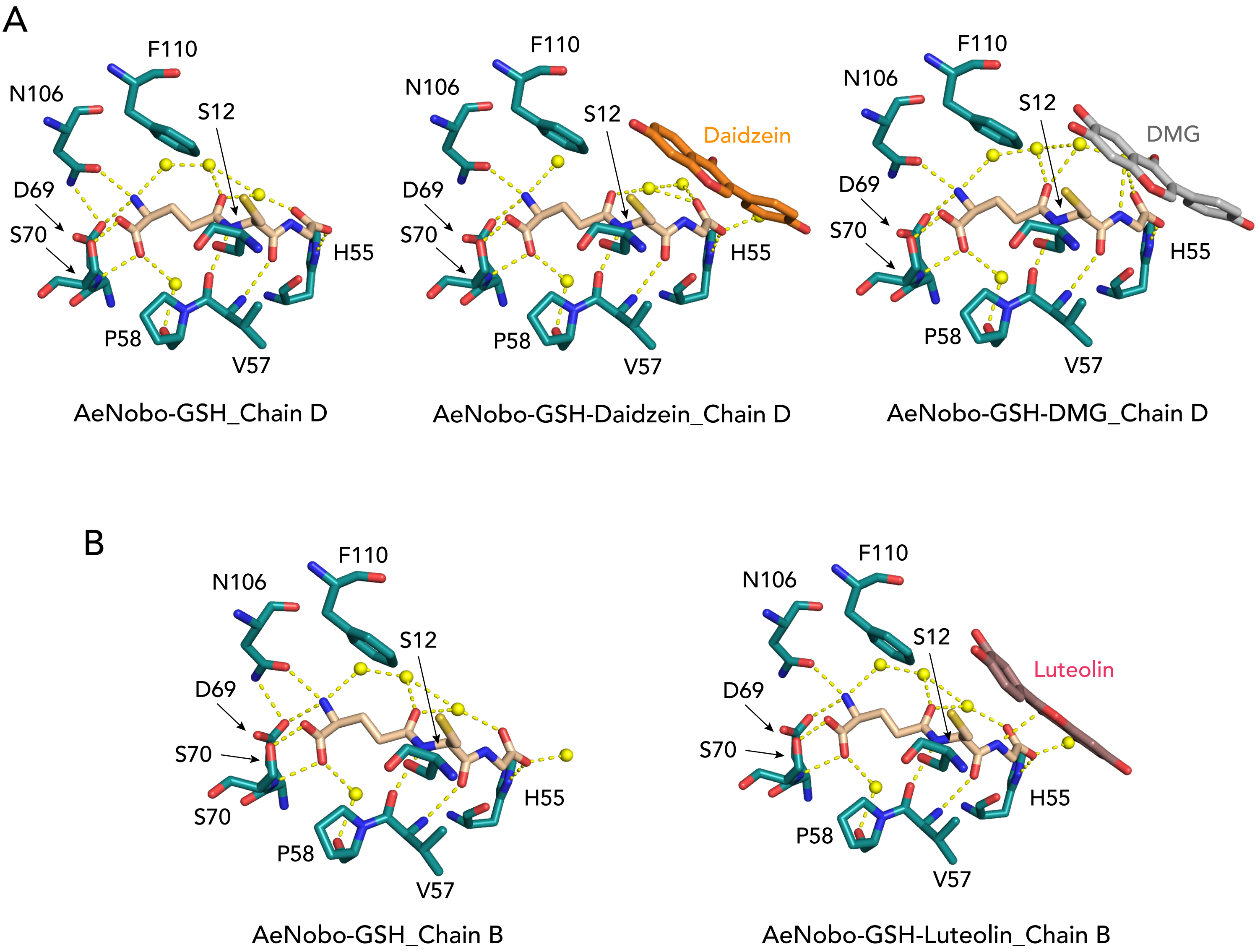
A
F110
N106
S12
D69
S70
H55
P58
V57
AeNobo-GSH_Chain D
Daidzein
AeNobo-GSH-Daidzein_Chain D
DMG
AeNobo-GSH-DMG_Chain D
B
AeNobo-GSH_Chain B
Luteolin
AeNobo-GSH-Luteolin_Chain B

Fig. S7

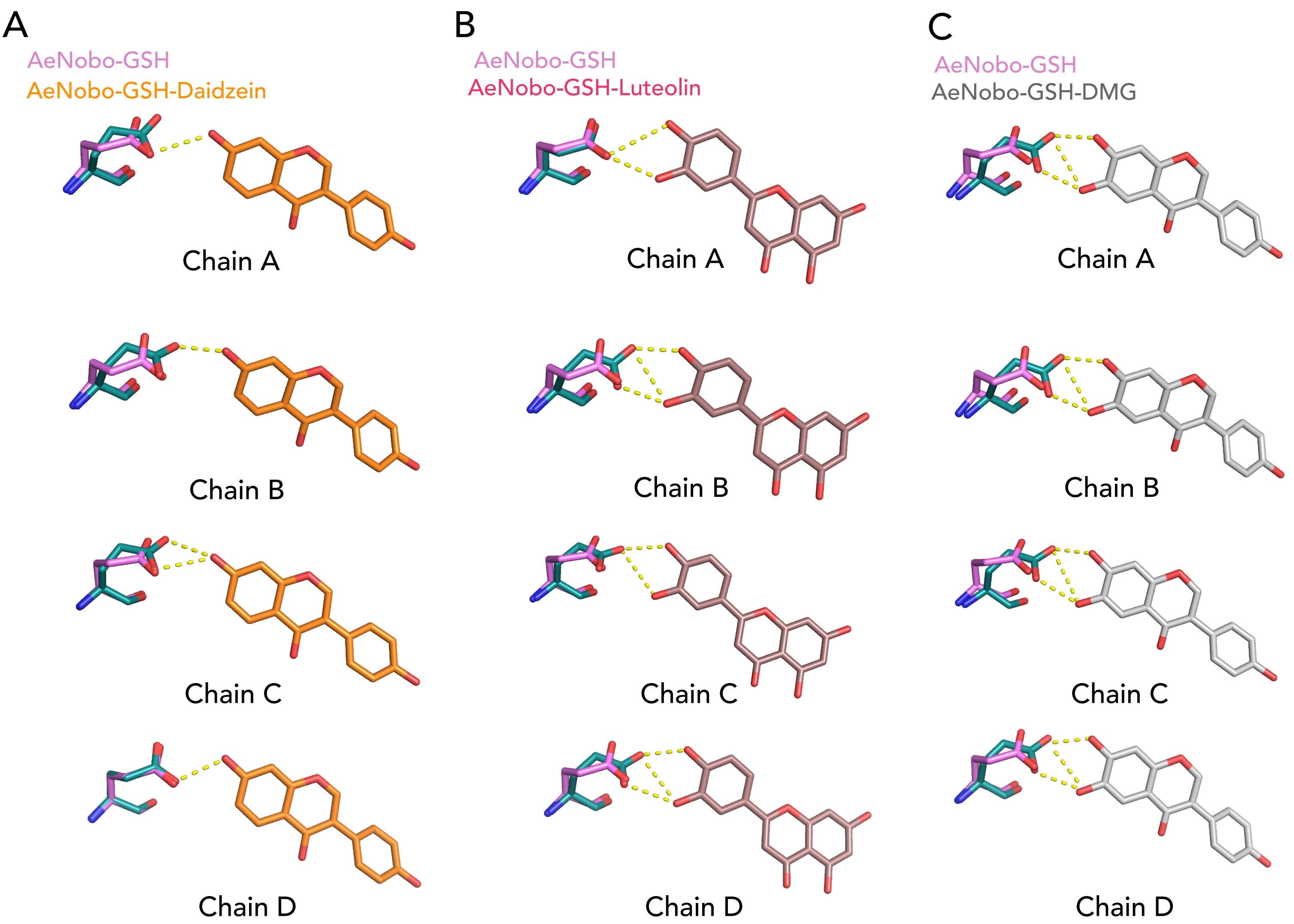

A
AeNobo-GSH
AeNobo-GSH-Daidzein
Chain A
Chain B
Chain C
Chain D
B
AeNobo-GSH
AeNobo-GSH-Luteolin
Chain A
Chain B
Chain C
Chain D
C
AeNobo-GSH
AeNobo-GSH-DMG
Chain A
Chain B
Chain C
Chain D

Fig. S8

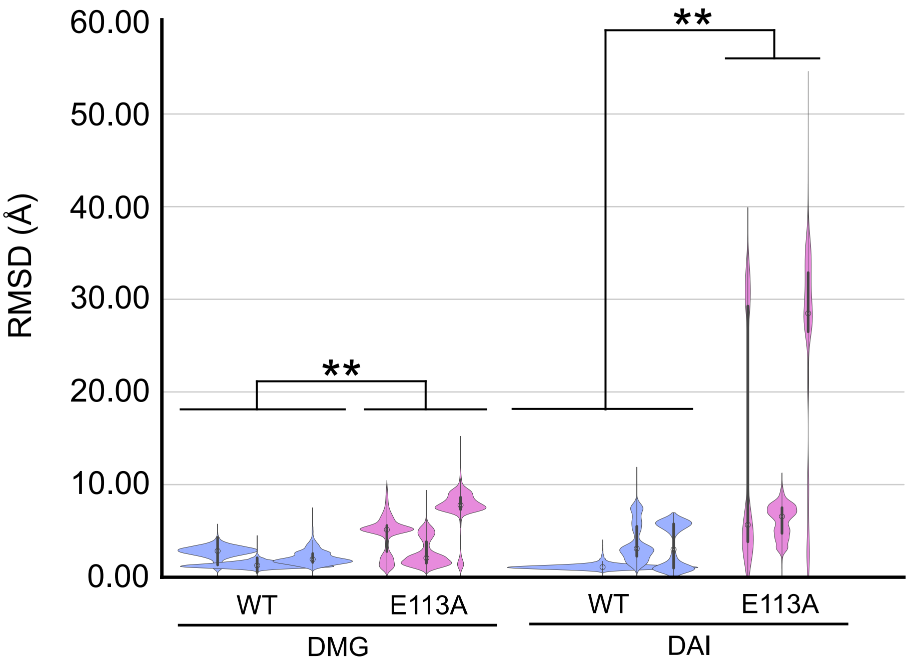

60.00
50.00
40.00
30.00
20.00
10.00
0.00
RMSD (Å)
**
**
WT
E113A
WT
E113A
DMG
DAI

Fig. S9

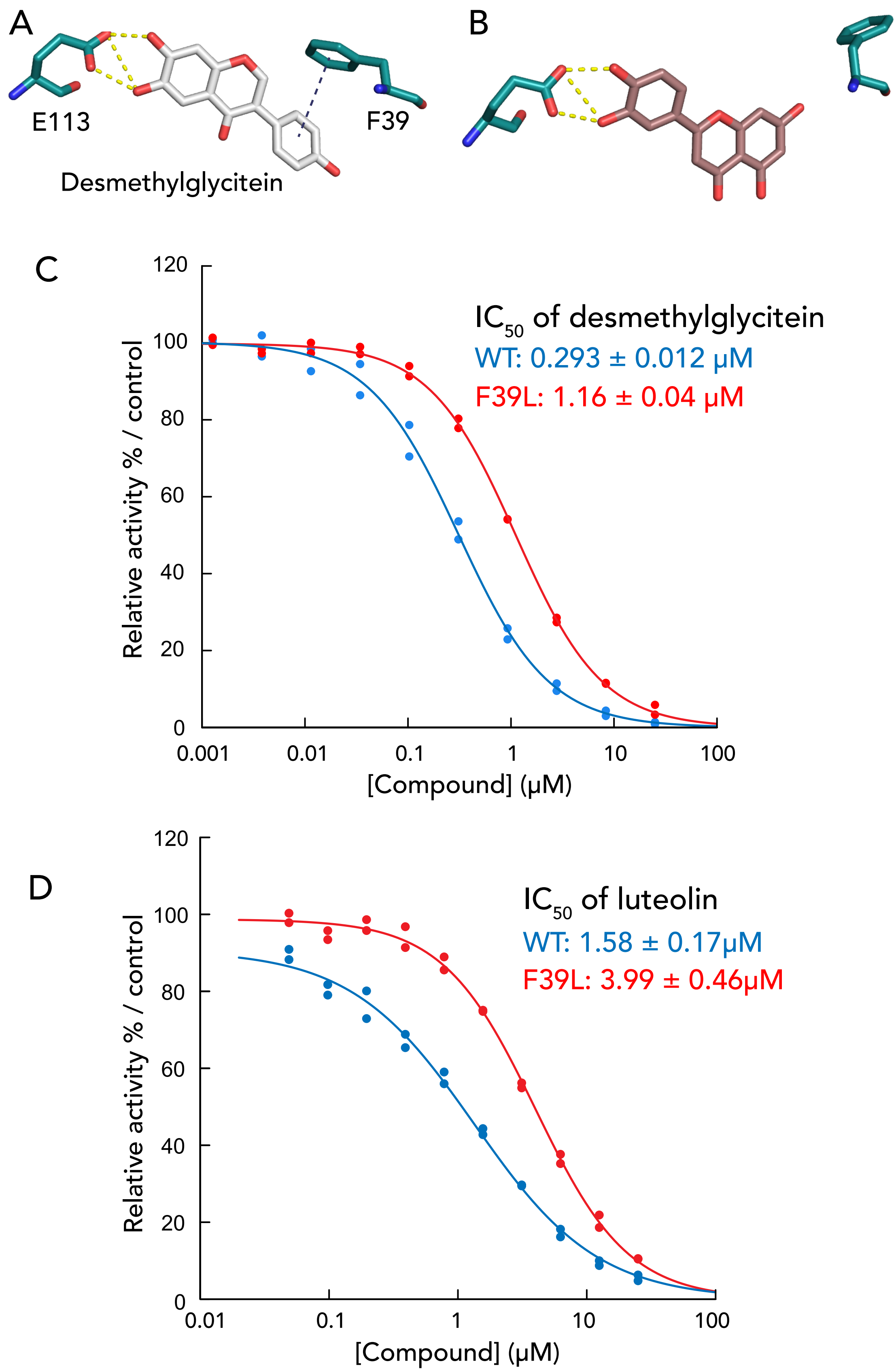
A
E113
F39
Desmethylglycitein
B
C
IC50 of desmethylglycitein
WT: 0.293 ± 0.012 μM
F39L: 1.16 ± 0.04 μM
Relative activity % / control
120
100
80
60
40
20
0
0.001
0.01
0.1
1
10
100
[Compound] (μM)
D
IC50 of luteolin
WT: 1.58 ± 0.17μM
F39L: 3.99 ± 0.46μM
Relative activity % / control
120
100
80
60
40
20
0
0.01
0.1
1
10
100
[Compound] (μM)

Fig. S10

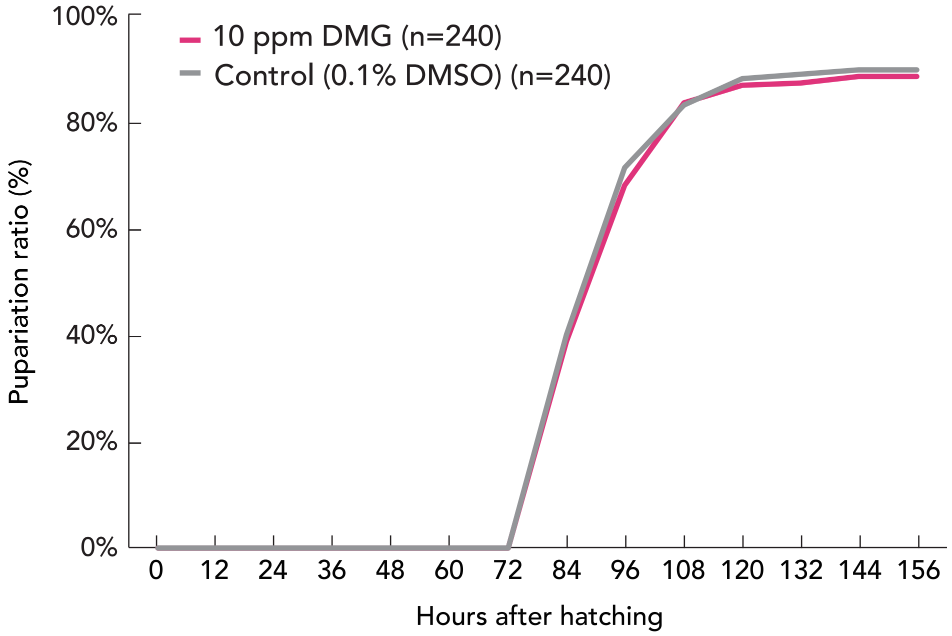
10 ppm DMG (n=240)
Control (0.1% DMSO) (n=240)
100%
80%
60%
40%
20%
0%
Pupariation ratio (%)
0
12
24
36
48
60
72
84
96
108
120
132
144
156
Hours after hatching
