# Supplementary material for: Molecular action of larvicidal flavonoids on ecdysteroidogenic glutathione S-transferase Noppera-bo in Aedes aegypti
Source: BMC Biol. 2022 Feb 17;20:43. doi: 10.1186/s12915-022-01233-2 (PMC8851771; doi:10.1186/s12915-022-01233-2)
Supplement: Supplementary file 2 — Additional file 2 : Table S1. Viability of noboKO animals expressing AeNobo. Table S2. Inhibitory activity of estrogenic compounds against AeNobo. Table S3. Crystallographic statistics. Table S4. RMSD (Å) of Cα atoms of the chains A/B/C/D. Table S5. Inhibitory activity of luteolin derivatives against AeNobo. Table S6. The head diameter of control and DMG-treated larvae. [file 12915_2022_1233_MOESM2_ESM.docx]

**Table S1. Viability of *nobo^KO^* animals expressing AeNobo.**

We scored the number of viable *nobo^KO^* / *nobo^KO^* adults. The transgene of *AeNobo* was driven by *phantom-GAL4* driver. *nobo^KO^ / CyO; UAS-AeNobo-WT* females were crossed with males of either *nobo^KO^ /CyO* (without any GAL4 transgene) or *nobo^KO^ /CyO; phm-GAL4/TM6c*. Genetic markers of the *CyO* and *TM6c* balancer chromosomes are *Cy-* and *Tb-*, respectively. *nobo^KO^* / *nobo^KO^* adults were *Cy+ Tb+*, while *nobo^KO^* heterozygous adults were *Cy- Tb-* or *Cy- Tb+*.

| GAL4 transgene | Number of adults |
| --- | --- |
| - | 0 (299) |
| *Phm-GAL4* | 35 (83) |

**Table S2. Inhibitory activity of estrogenic compounds against AeNobo.**

We selected 20 estrogenic compounds whose structures complexed with estrogen receptors are available in the Protein Data Bank (PDB; https://www.rcsb.org/). ‘No inhibition’ means that the IC_50_ value of a compound is greater than 25 μM. Among the examined chemicals, biochanin A, a flavonoid, is the only estrogenic chemical that inhibits the *in vitro* enzymatic activity of AeNobo. s.d.: standard deviation.

| Compound Name (ordered alphabetically) | PDB ID | IC_50_ (μM)  (mean ± s.d.) |
| --- | --- | --- |
| 2-Amino-1-methyl-6-phenylimidazo[4,5-b]pyridine | 2QXM | No inhibition |
| 1-benzyl 2-butyl benzene-1,2-dicarboxylate | 4MG6 | No inhibition |
| 16-benzylidene estrone | 5KRH | No inhibition |
| **Biochanin A** | 5JMM | **1.84 ± 0.06** |
| Biosphenol A (4,4'-propane-2,2-diyldiphenol) | 3UU7 | No inhibition |
| bis(2,4-dihydroxyphenyl)methanone | 4MGC | No inhibition |
| butyl 4-hydroxybenzoate | 4MG9 | No inhibition |
| 1,1-Dichloro-2,2-bis(4-chlorophenyl)ethene | 5KRA | No inhibition |
| diethylstilbestrol (4-[(*E*)-4-(4-hydroxyphenyl)hex-3-en-3-yl]phenol) | 4ZN7 | No inhibition |
| Ferutinine | 4MG7 | No inhibition |
| (*S*)-5-(4-hydroxy-3,5-dimethylphenyl)-1-methyl-2,3-dihydro-1H-inden-1-ol | 5TN4 | No inhibition |
| 4-[(1*S*,5*R*)-5-(hydroxymethyl)-8-methyl-3-oxabicyclo[3.3.1]non-7-en-2-yl]phenol | 2B1V | No inhibition |
| 4-[(1*R*,5*R*)-5-(hydroxymethyl)-6,8,9-trimethyl-3-oxabicyclo[3.3.1]non-7-en-2-yl]phenol | 1ZKY | No inhibition |
| 4-[(1*R*,5*R*)-4,4,8-trimethyl-3-oxabicyclo[3.3.1]non-7-en-2-yl]phenol | 2G44 | No inhibition |
| propyl 4-hydroxybenzoate | 4TV1 | No inhibition |
| Resveratrol | 4PP6 | No inhibition |
| 4-[2,2,2-trichloro-1-(4-hydroxyphenyl)ethyl]phenol | 4MGD | No inhibition |
| 4-(2,4,4-trimethylpentan-2-yl)phenol | 4MGA | No inhibition |
| α-zearalanol | 4MG8 | No inhibition |
| Zearalenone | 5KRC | No inhibition |

**Table S3. Crystallographic statistics.**

Values in parentheses correspond to the highest resolution shell.

|  | AeNobo-GSH | AeNobo-GSH-daidzein |
| --- | --- | --- |
| Wavelength (Å) | 0.9800 | 1.0000 |
| Resolution range (Å) | 48.03 - 1.51(1.56 - 1.51) | 47.96 - 1.95  (2.02 - 1.95) |
| Space group | *I*4_1_22 | *I*4_1_22 |
| Unit cell parameters  a, b, c (Å)  α, β, γ (°) | 151.88, 151.88, 147.30 90.00, 90.00, 90.00 | 152.21, 152.21, 146.83  90.00, 90.00, 90.00 |
| Total reflections | 3,632,980 (181,778) | 1,643,784 (168,410) |
| Unique reflections | 132,068 (12,907) | 62,161 (6,070) |
| Multiplicity | 27.5 (28.4) | 26.4 (27.7) |
| Completeness (%) | 98.93 (97.85) | 98.64 (98.55) |
| Mean I/sigma(I) | 21.3 (2.20) | 22.00 (4.01) |
| Wilson *B*-factor (Å^2^) | 17.37 | 26.11 |
| *R*_merge_ | 0.113 (2.06) | 0.120 (1.06) |
| *R*_pim_ | 0.022 (0.388) | 0.024 (0.202) |
| Reflections used in refinement | 132,046 (12,906) | 61,784 (6,067) |
| Reflections used for *R*_free_ | 6,770 (652) | 3,150 (286) |
| *R*_work_ | 0.186 | 0.190 |
| *R*_free_ | 0.208 | 0.242 |
| Number of non-hydrogen atoms | 7,851 | 7,680 |
| macromolecules | 6,887 | 6,755 |
| ligands | 89 | 164 |
| solvent | 875 | 761 |
| Protein residues | 882 | 876 |
| RMS (bonds, Å) | 0.007 | 0.008 |
| RMS (angles, °) | 0.90 | 0.98 |
| Ramachandran favored (%) | 97.03 | 96.76 |
| Ramachandran allowed (%) | 2.97 | 3.24 |
| Ramachandran outliers (%) | 0.00 | 0.00 |
| Rotamer outliers (%) | 0.14 | 0.14 |
| Clash score | 2.25 | 4.06 |
| Average *B*-factor (Å^2^) | 23.7 | 28.1 |
| macromolecules (Å^2^) | 22.7 | 27.4 |
| ligands (Å^2^) | 18.6 | 28.9 |
| solvent (Å^2^) | 32.8 | 33.5 |
| PDB ID | 7EBT | 7EBU |

(To be continued to the next page)

**Table S3, continued.**

|  | AeNobo-GSH-luteolin | AeNobo-GSH-DMG |
| --- | --- | --- |
| Wavelength (Å) | 1.0000 | 1.0000 |
| Resolution range (Å) | 36.69 - 1.50  (1.55 - 1.50) | 47.68 - 1.94  (2.01 - 1.94) |
| Space group | *I*4_1_22 | *I*4_1_22 |
| Unit cell parameters  a, b, c (Å)  α, β, γ (°) | 151.70, 151.70, 146.80  90.00 90.00 90.00 | 151.31, 151.31, 146.15  90.00, 90.00, 90.00 |
| Total reflections | 3,625,439 (368,971) | 1,652,478 (164,221) |
| Unique reflections | 135,312 (13,370) | 61,339 (5,941) |
| Multiplicity | 26.8 (27.6) | 26.9 (27.6) |
| Completeness (%) | 99.98 (99.99) | 98.03 (96.87) |
| Mean I/sigma(I) | 35.54 (3.17) | 26.25 (2.48) |
| Wilson *B*-factor (Å^2^) | 18.49 | 28.33 |
| *R*_merge_ | 0.0682 (1.28) | 0.145 (1.88) |
| *R*_pim_ | 0.0134 (0.247) | 0.0283 (0.360) |
| Reflections used in refinement | 135,302 (13,368) | 61,327 (5,941) |
| Reflections used for *R*_free_ | 6,767 (658) | 2,960 (304) |
| *R*_work_ | 0.186 | 0.172 |
| *R*_free_ | 0.208 | 0.219 |
| Number of non-hydrogen atoms | 7,838 | 7,670 |
| macromolecules | 6,802 | 6,763 |
| ligands | 192 | 168 |
| solvent | 844 | 739 |
| Protein residues | 875 | 875 |
| RMS (bonds, Å) | 0.007 | 0.008 |
| RMS (angles, °) | 0.96 | 0.96 |
| Ramachandran favored (%) | 97.33 | 97.81 |
| Ramachandran allowed (%) | 2.67 | 2.19 |
| Ramachandran outliers (%) | 0.00 | 0.00 |
| Rotamer outliers (%) | 0.00 | 0.14 |
| Clash score | 3.06 | 3.01 |
| Average *B*-factor (Å^2^) | 24.5 | 31.7 |
| macromolecules (Å^2^) | 23.4 | 31 |
| ligands (Å^2^) | 25.9 | 27.6 |
| solvent (Å^2^) | 32.7 | 38.4 |
| PDB ID | 7EBV | 7EBW |

**Table S4. RMSD (Å) of Cα atoms of the chains A/B/C/D.**

|  | Chain A | Chain B | Chain C | Chain D |
| --- | --- | --- | --- | --- |
| AeNobo-GSH-Daidzein | 0.109 | 0.115 | 0.104 | 0.115 |
| AeNobo-GSH-Luteolin | 0.049 | 0.056 | 0.067 | 0.048 |
| AeNobo-GSH-THI | 0.106 | 0.12 | 0.113 | 0.107 |

**Table S5. Inhibitory activity of luteolin derivatives against AeNobo.**

Luteolin derivatives used in this study are illustrated in Fig. 3*B*. In addition to the IC_50_ values, this table shows the presence of the hydroxyl residues (-OH) at the carbon positions of flavone nuclei. For example, luteolin possesses hydroxyl residues at C3', C4', C5, and C7 positions of the flavone nuclei. ‘No inhibition’ means that the IC50 value of a compound is greater than 25 μM. s.d.: standard deviation.

| Compound | C3' | C4' | C5 | C7 | IC_50_ (μM)  (mean ± s.d.) |
| --- | --- | --- | --- | --- | --- |
| Luteolin | -OH | -OH | -OH | -OH | 3.99 ± 0.46 |
| 5,3’,4’-Trihydroxyflavone | -OH | -OH | -OH |  | 1.64 ± 0.10 |
| 7,3’,4’-Trihydroxyflavone | -OH | -OH |  | -OH | 10.3 ± 0.47 |
| Apigenin |  | -OH | -OH | -OH | No inhibition |
| Chrysin |  |  | -OH | -OH | No inhibition |

**Table S6. The head diameter of control and DMG-treated larvae.**

The transverse diameter of the *Ae. aegypti* larval head was measured 24 h after adding 2.5 ppm DMG or control 0.1% DMSO. The distribution of these data is illustrated in Figure 5C. The values less than 0.3 are marked by bold.

| Control larvae (mm) | | | DMG-treated larvae (mm) | | |
| --- | --- | --- | --- | --- | --- |
| **0.21** | 0.4 | 0.43 | **0.21** | **0.24** | 0.36 |
| **0.21** | 0.41 | 0.43 | **0.21** | **0.24** | 0.36 |
| **0.22** | 0.41 | 0.43 | **0.21** | **0.25** | 0.36 |
| **0.23** | 0.41 | 0.44 | **0.22** | **0.25** | 0.36 |
| 0.3 | 0.41 | 0.44 | **0.22** | **0.25** | 0.37 |
| 0.33 | 0.41 | 0.44 | **0.22** | **0.25** | 0.38 |
| 0.34 | 0.41 | 0.45 | **0.23** | **0.25** | 0.39 |
| 0.37 | 0.41 | 0.45 | **0.23** | **0.25** | 0.39 |
| 0.37 | 0.41 | 0.45 | **0.23** | **0.25** | 0.39 |
| 0.37 | 0.41 | 0.46 | **0.23** | **0.26** | 0.39 |
| 0.39 | 0.42 | 0.6 | **0.23** | **0.28** | 0.4 |
| 0.39 | 0.42 | 0.6 | **0.23** | 0.33 | 0.4 |
| 0.39 | 0.42 |  | **0.24** | 0.33 | 0.41 |
| 0.39 | 0.42 |  | **0.24** | 0.34 | 0.41 |
| 0.39 | 0.42 |  | **0.24** | 0.34 | 0.41 |
| 0.4 | 0.42 |  | **0.24** | 0.34 | 0.42 |
| 0.4 | 0.43 |  | **0.24** | 0.34 | 0.44 |
| 0.4 | 0.43 |  | **0.24** | 0.35 | 0.45 |
| 0.4 | 0.43 |  | **0.24** | 0.36 | 0.68 |
| Average: 0.40 mm | | | Average: 0.31 mm | | |
| # of less than 0.3 mm: 4 | | | # of less than 0.3 mm: 30 | | |
| # of total larvae: 50 | | | # of total larvae: 57 | | |
